# A random effects meta-analysis model with Box-Cox transformation

**DOI:** 10.1186/s12874-017-0376-7

**Published:** 2017-07-19

**Authors:** Yusuke Yamaguchi, Kazushi Maruo, Christopher Partlett, Richard D. Riley

**Affiliations:** 1 Japan-Asia Data Science, Development, Astellas Pharma Inc., 2-5-1, Nihonbashi-Honcho, Chuo-ku, Tokyo, 103-8411 Japan; 2 Department of Clinical Epidemiology, National Center of Neurology and Psychiatry, 4-1-1, Ogawahigashi-cho, Kodaira, Tokyo, 187-8551 Japan; 3 National Perinatal Epidemiology Unit, University of Oxford, Oxford, OX1 2JD UK; 4 Research Institute for Primary Care and Health Sciences, Keele University, Staffordshire, ST5 5BG UK

**Keywords:** Meta-analysis, Random effects model, Skewed data, Box-Cox transformation

## Abstract

**Background:**

In a random effects meta-analysis model, true treatment effects for each study are routinely assumed to follow a normal distribution. However, normality is a restrictive assumption and the misspecification of the random effects distribution may result in a misleading estimate of overall mean for the treatment effect, an inappropriate quantification of heterogeneity across studies and a wrongly symmetric prediction interval.

**Methods:**

We focus on problems caused by an inappropriate normality assumption of the random effects distribution, and propose a novel random effects meta-analysis model where a Box-Cox transformation is applied to the observed treatment effect estimates. The proposed model aims to normalise an overall distribution of observed treatment effect estimates, which is sum of the within-study sampling distributions and the random effects distribution. When sampling distributions are approximately normal, non-normality in the overall distribution will be mainly due to the random effects distribution, especially when the between-study variation is large relative to the within-study variation. The Box-Cox transformation addresses this flexibly according to the observed departure from normality. We use a Bayesian approach for estimating parameters in the proposed model, and suggest summarising the meta-analysis results by an overall median, an interquartile range and a prediction interval. The model can be applied for any kind of variables once the treatment effect estimate is defined from the variable.

**Results:**

A simulation study suggested that when the overall distribution of treatment effect estimates are skewed, the overall mean and conventional *I* ^2^ from the normal random effects model could be inappropriate summaries, and the proposed model helped reduce this issue. We illustrated the proposed model using two examples, which revealed some important differences on summary results, heterogeneity measures and prediction intervals from the normal random effects model.

**Conclusions:**

The random effects meta-analysis with the Box-Cox transformation may be an important tool for examining robustness of traditional meta-analysis results against skewness on the observed treatment effect estimates. Further critical evaluation of the method is needed.

**Electronic supplementary material:**

The online version of this article (doi:10.1186/s12874-017-0376-7) contains supplementary material, which is available to authorized users.

## Background

Meta-analysis is a useful statistical tool for combining results from independent studies, for example where estimates of a treatment effect (e.g odds ratio, mean difference or standardised mean difference) from randomised controlled trials are pooled in order to make inferences about an overall summary effect. A random effects meta-analysis model that assumes different true treatment effects underlying different studies is often needed as it allows for unexplained heterogeneity across studies [1]. In the random effects model, the true treatment effects for each study are usually assumed to follow a normal distribution; thus, an overall mean (summary) effect is obtained by estimating the mean parameter of this distribution.

In this article, we focus on problems caused by an inappropriate normality assumption of the random effects distribution, in particular in regard to the impact on the mean effect estimate, quantification of heterogeneity and prediction interval. Turner et al. [2] suggested that the misspecification of the random effects distribution seriously affected the estimates of the random effects variances. Lee and Thompson [3] showed that the shape of the predictive distributions of the treatment effect was substantially affected by the shape of the assumed random effects distribution. The normality assumption may therefore be a restrictive assumption for meta-analysts who are interested in producing a summary treatment effect, quantifying heterogeneity and deriving a prediction interval, especially if the true random effects distribution is skewed.

Alternative parametric distributions have been considered for the random effects distribution in mixed models; for example, t-distribution [4], gamma or mirrored gamma distribution [2], Laplace (double-exponential) distribution [5], skewed normal or skewed t-distribution [3], mixture distributions [6]. And also, as an approach to outliers in meta-analysis, Baker and Jackson [7] proposed a model that allows the random effects to be long-tailed, which provides a down-weighting of outliers and removes the necessity for an arbitrary decision to exclude the outliers. Gumedze and Jackson [8] used likelihood ratio test statistics to detect and down-weight outliers in the meta-analysis. However, each has disadvantages as discussed in Lee and Thompson [3]; for example, the mixture distributions can fail in situations where there are a few outliers. When assuming a skewed distribution for the random effects in a meta-analysis, the mean and the variance are not appropriate representatives for summarising the skewed true treatment effects. The overall mean for the skewed treatment effects would be pulled in the direction of the extreme observed estimates; hence, it could result in misleading conclusions from the meta-analysis. It is also not straightforward to quantify the impact of heterogeneity, such as *I* ^2^, if there is a non-normal random effects distribution. Indeed, Higgins et al. [9] mentioned that some alternative parametric distributions may not have parameters that naturally describe an overall effect, or the heterogeneity across studies.

Here, we propose a novel random effects meta-analysis model, where a Box-Cox transformation [10] is applied to the observed treatment effect estimates. The aim of the Box-Cox transformation is to achieve approximate normality of the overall distribution of the observed treatment effect estimates after transformation. The use of the Box-Cox transformation in linear models has been studied extensively [11–14]. In particular, Gurka et al. [15] provided an extension of the Box-Cox transformation to linear mixed models and demonstrated that a single transformation parameter would simultaneously help achieve normality of both the random effects and the residual error. However, the Box-Cox transformation has not been used commonly in the context of meta-analysis. Indeed, a work by Kim et al. [16] is the only meta-analytic application of the Box-Cox transformation that we are aware of. They proposed a multivariate response Box-Cox regression model for modelling individual patient data (IPD). However, the approach by Kim et al. [16] cannot apply to the cases of more readily available aggregate data (such as observed estimates of the treatment effect and their standard errors), because their model just allows the individual patient responses to be transformed and thus requires IPD. We rather consider transforming the observed treatment effect estimates using the Box-Cox transformation and suggest summarising the overall effect by an overall median rather than the overall mean, and quantifying the impact of heterogeneity by an interquartile range rather than commonly used *I* ^2^. The method no longer requires the IPD.

In this section, we introduce two motivating examples which will be used for illustrating the proposed model. In the “Methods” section, we introduce the standard normal random effects models, and describe how to make the Bayesian inference in the random effects meta-analysis from the following viewpoints: the overall mean effect, the heterogeneity and the prediction interval. And then, we describe our new random effects model with the Box-Cox transformation. In the “Results” section, we conduct a simulation study to examine the performance of our proposed model under some situations where true random effects follow non-normal distributions, and compare the results with those from the standard normal random effects model. Moreover, we illustrate our proposed model using the examples. Finally, we conclude this article with some discussion.

### Motivating examples

#### Example 1: Teacher expectancy on pupil IQ

**Fig. 1:**
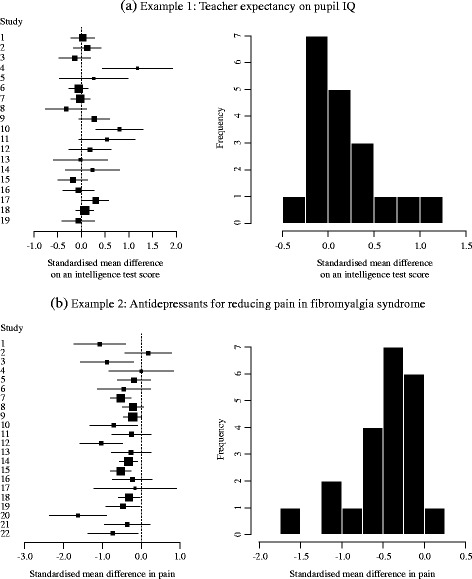
Forest plot and histogram. **a** 19 experiments investigating teacher expectancy on pupil IQ, **b** 22 studies investigating antidepressants for reducing pain in fibromyalgia syndrome

Raudenbush [17] reviewed randomised experiments of the effects of teacher expectancy on pupil IQ (see also Raudenbush and Bryk [18] for the details). The research question was: do pupils have a better performance if their teacher expected them to perform well? In each of 19 experiments identified, after administering an intelligence test to a sample of students, a randomly selected portion of the students were identified to their teachers as “likely to experience substantial intellectual growth" (the treatment group). All students were tested again, and the standardised mean difference between the test scores of students in the treatment group and those of the other students was evaluated as a treatment effect. The data from the 19 experiments was obtained from Table 18.2 in Hartung et al. [19]. Figure 1a shows a forest plot and a histogram of the estimates of the standardised mean differences, with positive values indicating a higher mean score for the treatment (high-expectancy) group. Although the histogram is a slightly naive display because it ignores the different weighting (number of participants) in the studies, it does suggest the presence of positive skewness in the observed distribution of the estimates.

#### Example 2: Antidepressants for reducing pain in fibromyalgia syndrome

Hauser et al. [20] reported a meta-analysis of randomised controlled trials to investigate the efficacy of antidepressants for fibromyalgia syndrome, which is a chronic pain disorder associated with multiple debilitating symptoms. 22 trials using different classes of antidepressants were involved in the analysis, and estimates of the standardised mean difference in pain (for the antidepressant group minus the control group) were combined using a random effects model. The data was obtained from Figure 3 in Riley et al. [21]. Figure 1b shows a forest plot and a simple histogram of estimates of the standardised mean differences, with negative values indicating a benefit for the antidepressants. The histogram suggests the presence of negative skewness on the estimates.

## Methods

### Normal random effects model

We first consider the standard normal random effects model for a meta-analysis of *k* studies. Let *y*_*i*_ and $\sigma _{i}^{2}$ be an estimate of a treatment effect and its variance observed from the *i*th study (*i*=1,…,*k*), respectively. Then the normal random effects model is given by

1 $$\begin{array}{@{}rcl@{}} &y_{i}=\theta_{i}+\epsilon_{i},\\ &\theta_{i}=\theta+u_{i},\\ &\epsilon_{i}\sim N\left(0,\sigma_{i}^{2}\right),\quad u_{i}\sim N\left(0,\tau^{2}\right) \end{array} $$

where *θ*_*i*_ is the true (but unknown) treatment effect for the *i*th study and is represented by the sum of *θ* and *u*_*i*_. The *u*_*i*_ is assumed to follow a normal distribution with mean zero and variance *τ* ^2^, indicating that the true treatment effect for the *i*th study, *θ*_*i*_, is normally distributed about *θ* with the between-study variance *τ* ^2^. *ε*_*i*_ is a sampling error within the *i*th study and is assumed to follow a normal distribution with mean zero and variance $\sigma _{i}^{2}$, where the within-study variance $\sigma _{i}^{2}$ is commonly considered to be known. Of key interest is an estimate of the mean parameter of the random effects distribution, *θ*, as this provides the mean treatment effect of the included studies. Also of interest is an estimate of *τ* ^2^, to quantify the amount of heterogeneity and to derive a 95 percent prediction interval [9].

#### Bayesian estimation of model parameters

We here use a Bayesian approach for estimating parameters involved in the normal random effects model (1). Marginalising the true treatment effect (*θ*_*i*_) from a joint distribution of *y*_*i*_ and *θ*_*i*_, we have *y*_*i*_ $y_{i}\sim N\left (\theta,\tau ^{2}+\sigma _{i}^{2}\right)$. Given *θ* and *τ* ^2^, the conditional density function of *y*=(*y*_1_,…,*y*_*k*_) is written as

$$\begin{array}{@{}rcl@{}} p(y|\theta,\tau^{2})=\prod\limits_{i=1}^{k}\frac{1}{\sqrt{2\pi}\left(\tau^{2}+\sigma_{i}^{2}\right)^{1/2}}\exp\left \{-\frac{(y_{i}-\theta)^{2}}{2\left(\tau^{2}+\sigma_{i}^{2}\right)}\right \}. \end{array} $$

Then a posterior distribution of *θ* and *τ* ^2^ can be given as

$$\begin{array}{@{}rcl@{}} p\left(\theta,\tau^{2}|y\right)\propto p\left(y|\theta,\tau^{2}\right)p\left(\theta,\tau^{2}\right) \end{array} $$

where *p*(*θ*,*τ* ^2^) is a prior density for *θ* and *τ* ^2^. Since minimally informative prior distributions are appropriate in the absence of definite priori information, we here use the following vague priors:

2 $$\begin{array}{@{}rcl@{}} \theta&\sim&N(0,10000),\\ \tau&\sim&U(0,b) \end{array} $$

where *b* is a constant value given by practitioners. It is well known that the results from Bayesian meta-analyses could be potentially sensitive to the choice of prior distributions, especially to the prior of the between-study variance *τ* ^2^ (e.g. see Lambert et al. [22] for the details). Various non-informative priors for *τ* ^2^ have been suggested in previous researches; for example, a uniform prior on *τ* [23, 24], a uniform prior on log(*τ* ^2^) [25], an inverse-gamma prior on *τ* ^2^ [26] and a half-Cauchy prior on *τ* [24]. We consider the uniform prior on *τ* in the range of (0,*b*), where the upper limit, *b*, should be decided according to the individual situations. The uniform prior on the standard deviation increasingly becomes known as a reasonable alternative to a more general inverse-gamma prior on variance (e.g. see Gelman [24] for the details). In practice the sensitivity of specified priors should be investigated by applying many other priors for the parameters or by using prior distribution based on empirical evidence [27, 28], though we in this article avoid the extensive discussion for the prior. In the Bayesian framework, a posterior mean and a 95 percent credible interval are commonly used for summarising the posterior distribution. We implement our Bayesian analysis by using Markov chain Monte Carlo (MCMC) methods, with a free R software and its rstan package (see the Stan Modelling Language User’s Guide and Reference Manual [29] for the details). The source code for conducting meta-analyses with the normal random effects model (1) is shown in Additional file 1.

#### Quantification of heterogeneity

The magnitude of heterogeneity across studies can be quantified by the posterior estimate of the between-study variance *τ* ^2^ or its square root. In the Bayesian framework, we obtain the posterior distribution of *τ* ^2^ and its credible interval, which can be used for quantifying the magnitude of the between-study heterogeneity of the true treatment effects. However, the between-study variance may be sensitive to the metric of the treatment effect, and thus this is not necessarily appropriate for the purpose of comparing several meta-analyses in terms of the heterogeneity [30]. If we are interested in what proportion of the observed variance reflects real differences in the treatment effect, the *I* ^2^ proposed by Higgins and Thompson [30] is useful for this purpose. Under the normal random effects model (1), the *I* ^2^ is expressed as a function of *τ* ^2^, given by

3 $$\begin{array}{@{}rcl@{}} I^{2}=\frac{\tau^{2}}{\tau^{2}+s^{2}} \end{array} $$

where

4 $$\begin{array}{@{}rcl@{}} s^{2}=\frac{(k-1)\sum_{i=1}^{k} 1/\sigma_{i}^{2}}{\left(\sum_{i=1}^{k} 1/\sigma_{i}^{2}\right)^{2}-\sum_{i=1}^{k} \left(1/\sigma_{i}^{2}\right)^{2}}. \end{array} $$

Here, *s* ^2^ is referred to as ‘typical’ within-study variance. In this article, we calculate *I* ^2^ based on the estimated *τ* ^2^ during each sample of the Bayesian estimation process; that is, we summarise the posterior distribution of *I* ^2^ derived by using samples of *τ* ^2^ drawn from its posterior distribution.

#### Prediction interval

In the Bayesian framework, a predictive distribution of the treatment effect in a new study is given by

5 $$\begin{array}{@{}rcl@{}} p(\theta_{\text{new}}|y)=\int\int p\left(\theta_{\text{new}}|\theta,\tau^{2}\right)p\left(\theta,\tau^{2}|y\right)d\theta d\tau^{2}. \end{array} $$

Following estimation of model (1) using the MCMC, a (100−*q*) percent prediction interval is obtained by taking (*q*/2)th and (100−*q*/2)th quantiles of samples drawn from the predictive distribution (5). For example, lower and upper bounds of 95 percent prediction interval are given by 2.5th and 97.5th quantiles of samples from the predictive distribution, respectively. Note that this is just one option for obtaining the 95 percent prediction interval, and other ways of defining the interval can be chosen depending on where we want to take the lower and upper limits. When interest lies in predicting probability that the treatment is effective by more than a clinically important difference in a new study, we can find this by calculating the proportion of samples drawn from the predictive distribution which satisfy a specified criteria for the effectiveness of the treatment (e.g. odds ratio < 80 percent). The sampling can be achieved by first drawing samples of parameters from the posterior distribution *p*(*θ*,*τ* ^2^|*y*) and then drawing samples from *p*(*θ*_new_|*θ*,*τ* ^2^) with fixed parameters obtained in the previous step [4]. In the second step, the drawing is performed by *θ*_new_∼*N*(*θ*,*τ* ^2^). In this manner, the prediction interval accounts for the heterogeneity in true treatment effects and naturally incorporates all parameter uncertainty (e.g. in *θ* and *τ* ^2^). It should be interpreted differently from the credible interval for the mean effect, which only indicates the uncertainty in the mean effect itself, not the entire distribution of true treatment effects across studies [21].

### Random effects model with Box-Cox transformation

#### Box-Cox transformation

Before giving our proposed model, we first introduce the Box-Cox transformation for a standard consideration of a continuous variable. The aim of the Box-Cox transformation is to achieve approximate normality of a variable (say, *y*_*i*_) after transformation [10]. Roughly saying, it can be used for changing scale of data so that the transformed data are distributed symmetrically. In particular, we consider a normalised shift transformation given by

6 $$ y_{i}(\lambda,\alpha)= \left\{ \begin{aligned} &{\frac{(y_{i}+\alpha)^{\lambda}-1}{\lambda\dot{g}(\alpha)^{\lambda-1}}},\qquad \lambda \neq 0 \\ &\log(y_{i}+\alpha)\dot{g}(\alpha),\quad\, \lambda = 0 \\ \end{aligned} \right.  $$

for *y*_*i*_+*α*>0 (*i*=1,…,*k*), where we keep *y*_*i*_ for ease of notation, though *y*_*i*_ could refer to any continuous measure (not just an effect size). *λ* and *α* denote a transformation and a shift parameter respectively, and these parameters are estimated from the observed data. $\dot {g}(\alpha)$ is a geometric mean of *y*_*i*_+*α* for *i*=1,…,*k*. The normalisation using the geometric mean $\dot {g}(\alpha)$ could lead a stable estimation of *λ* and *α*, in comparison with a standard Box-Cox transformation without the normalisation.

To be exact, it is proper to assume that the transformed variable *y*_*i*_(*λ*,*α*) follows a truncated normal distribution except for the case of *λ*=0, because of the condition that *y*_*i*_+*α* must be a positive value. When interest lies in inference in original scale before transformation (not in the scale after transformation), we need to specify the distribution of the observed values before the Box-Cox transformation and deal with the truncation precisely [31–34]. However, these are beyond the scope of this article. For mathematical convenience, we below assume that the transformed variable *y*_*i*_(*λ*,*α*) follows a normal distribution with no consideration of the truncation.

#### Proposed meta-analysis model and its estimation

Let *y*_*i*_’s be the treatment effect estimates (e.g. log odds ratio or mean difference) from the available studies in a meta-analysis. We propose the following random effects model for the Box-Cox transformed *y*_*i*_:

7 $$\begin{array}{@{}rcl@{}} y_{i}(\lambda,\alpha)&=&\mu_{i}+\epsilon_{i},\\ \mu_{i}&=&\mu+u_{i},\\ \epsilon_{i}\sim N\left(0,\phi_{i}^{2}(\lambda,\alpha)\right),&&u_{i}\sim N(0,\tau^{2}). \end{array} $$

The model structure is basically same as the normal random effects model (1), though now the Box-Cox transformation (6) is applied to the observed treatment effect estimates for each study and *μ*_*i*_ denotes a true effect of the Box-Cox transformed variable *y*_*i*_(*λ*,*α*) which has a ‘known’ variance of $\phi _{i}^{2}(\lambda,\alpha)$ (see section below). The proposed model aims to improve the overall normality of the observed treatment effects estimates (*y*_*i*_) across studies; their overall distribution is the sum of the random effects distribution of true effects and the within-study sampling distribution of estimates. As long as the studies have reasonable sample size, the within-study sampling distribution of *y*_*i*_ will be approximately normal due to the central limit theorem. However, there is no such guarantee for the random effects distribution [7], and thus any asymmetry in the random effects distribution will consequently cause asymmetry in the overall distribution for *y*_*i*_. The following processes are required to implement the proposed random effects model (7).

##### Definition of variance of the Box-Cox transformed treatment effect estimate

In the proposed model (7), the variance of the Box-Cox transformed treatment effect estimate, $\phi _{i}^{2}(\lambda,\alpha)$; i.e. the variance of *y*_*i*_(*λ*,*α*) given *μ*_*i*_, must be defined. Since the variance needs to be assigned for each study separately, we here approximate the variance of *y*_*i*_ by a first order Taylor series about *y*_*i*_(*λ*,*α*)=*E*[*y*_*i*_(*λ*,*α*)] as follows:

$$\begin{array}{@{}rcl@{}} {{} \begin{aligned} V[y_{i}]&\approx V[y_{i}(\lambda,\alpha)]\left\{\left.\frac{\partial y_{i}}{\partial y_{i}(\lambda,\alpha)}\right|_{y_{i}(\lambda,\alpha)=E[y_{i}(\lambda,\alpha)]}\right\}^{2}\\ &=\left\{ \begin{array}{ll} V[y_{i}(\lambda,\alpha)]\dot{g}(\alpha)^{2\lambda-2}\left\{\lambda\dot{g}(\alpha)^{\lambda-1}E[y_{i}(\lambda,\alpha)]+1\right\}^{2/\lambda-2}, & \lambda \neq 0 \\ {\frac{V[y_{i}(\lambda,\alpha)]}{\dot{g}(\alpha)^{2}}}\exp\left\{ {\frac{2E[y_{i}(\lambda,\alpha)]}{\dot{g}(\alpha)}}\right\}, & \lambda = 0 \\ \end{array} \right.. \end{aligned}} \end{array} $$

For ${V\,[\!y_{i}]=\sigma _{i}^{2}}$, *E* [ *y*_*i*_(*λ*,*α*)]=*μ* and ${V\,[\!y_{i}(\lambda,\alpha)]=\phi _{i}^{2}} (\lambda,\alpha)$, we have an approximation of the variance of the Box-Cox transformed treatment effect estimate, written by

8 $$  \phi_{i}^{2}(\lambda,\alpha)\approx \left\{ \begin{aligned} &{\frac{\sigma_{i}^{2}}{\dot{g}(\alpha)^{2\lambda-2}}}\left\{\lambda\dot{g}(\alpha)^{\lambda-1}\mu+1\right\}^{2-2/\lambda},\quad \lambda \neq 0 \\ &\sigma_{i}^{2}\dot{g}(\alpha)^{2}\exp\left\{ {-\frac{2\mu}{\dot{g}(\alpha)}}\right\}, \qquad\qquad\quad\, \lambda = 0 \\ \end{aligned} \right.  $$

where recall *α* is the shift parameter, *λ* is the transformation parameter, $\dot {g}(\alpha)$ is the geometric mean of *y*_*i*_+*α* for *i*=1,…,*k*, *μ* is the mean parameter of the random effects distribution in the transformed scale and $\sigma _{i}^{2}$ is the within-study variance from the *i*th study. The relationship between variances before and after transformation has been applied for stabilising variance [35, 36] or representing inhomogeneity variances in linear models with Box-Cox transformation weighting [37].

##### Frequentist estimation of *λ* and *α*

We treat the transformation parameter *λ* and the shift parameter *α* as non-stochastics; i.e. we first estimate these parameters by a maximum likelihood estimation, and then make inference about the other parameters *μ* and *τ* ^2^ conditioning on $\lambda =\hat {\lambda }$ and $\alpha =\hat {\alpha }$, where $\hat {\lambda }$ and $\hat {\alpha }$ are maximum likelihood estimates of *λ* and *α* respectively. Maruo and Goto [34] has investigated the influence of not considering the uncertainty associated with estimation of *λ*, and showed the confidence interval around the median from an univariate analysis with the Box-Cox transformation was slightly liberal (from two to three percent).

A log likelihood function for (*μ*,*τ* ^2^,*λ*,*α*) is given by

9 $$ \begin{aligned} l(\mu,\tau^{2},\lambda,\alpha)&=\sum\limits_{i=1}^{k}\left[\vphantom{\frac{\left(y_{i}(\lambda,\alpha)-\mu\right)^{2}}{2(\tau^{2}+\phi^{2}_{i}(\lambda,\alpha))}}-\frac{1}{2}\log\left(\tau^{2}+\phi^{2}_{i}(\lambda,\alpha)\right)\right.\\ &\quad-\left.\frac{\left(y_{i}(\lambda,\alpha)-\mu\right)^{2}}{2(\tau^{2}+\phi^{2}_{i}(\lambda,\alpha))}\right]. \end{aligned}  $$

A grid search procedure is one simple approach for finding $\hat {\lambda }$ and $\hat {\alpha }$ which maximises the log likelihood (9) with respect to *λ* and *α*. For a large set of values for (*λ*,*α*), the log likelihood can be rewritten as *l*(*μ*,*τ* ^2^,*λ*,*α*)=*l*_*λ*,*α*_(*μ*,*τ* ^2^) where *μ* and *τ* ^2^ vary but *λ* and *α* are fixed. Maximising *l*_*λ*,*α*_(*μ*,*τ* ^2^) with respect to *μ* and *τ* ^2^, we obtain their estimates for the fixed *λ* and *α* as

$$\begin{array}{@{}rcl@{}} \left(\hat{\mu}(\lambda,\alpha),\hat{\tau}^{2}(\lambda,\alpha)\right)=\underset{\mu,\,\tau^{2}}{\mathrm{arg\,max}}\ l_{\lambda,\alpha}\left(\mu,\tau^{2}\right). \end{array} $$

Substituting the estimates ${\hat {\mu }(\lambda,\alpha)}$ and ${\hat {\tau }^{2}(\lambda,\alpha)}$ into *l*_*λ*,*α*_(*μ*,*τ* ^2^), then we have a log likelihood $l_{\lambda,\alpha }(\hat {\mu }(\lambda,\alpha)$, $\hat {\tau }^{2}(\lambda,\alpha))$ for the fixed *λ* and *α*. Then, we obtain a set of (*λ*,*α*) for which the log likelihood takes the largest value as the approximate values of $\hat {\lambda }$ and $\hat {\alpha }$.

An issue known as non-regular problem is caused in the maximum likelihood estimation of *α* because the range of the distribution is determined by the unknown shift parameter *α* [38, 39]. For example, it is argued that the likelihood function of *α* fails to have a local maximum [38]. In this article, we focus on the inference in the original scale before transformation; hence, we assume the concern about the estimation of *α* would not have substantial impact than if we were interested in the exact estimation of the transformation and the shift parameter (*λ* and *α*) themselves. This could be an area of further research.

##### Bayesian estimation of model parameters

Given $\hat {\lambda }$ and $\hat {\alpha }$ (i.e. optimum transformation of the treatment effect, $y_{i}(\hat {\lambda },\hat {\alpha })$ for *i*=1,…,*k* and their variances), we take a Bayesian approach to estimation of the unknown parameters from the Box-Cox meta-analysis model (7), *μ* and *τ* ^2^. Marginalising the true treatment effect (*μ*_*i*_) from a joint distribution of $y_{i}(\hat {\lambda },\hat {\alpha })$ and *μ*_*i*_, we have $y_{i}(\hat {\lambda },\hat {\alpha })\sim N(\mu,\tau ^{2}+\phi _{i}^{2}(\hat {\lambda },\hat {\alpha }))$. The posterior distribution of *μ* and *τ* ^2^ is given by

$$\begin{array}{@{}rcl@{}} p(\mu,\tau^{2}|y;\hat{\lambda},\hat{\alpha})\propto p(y|\mu,\tau^{2};\hat{\lambda},\hat{\alpha})p(\mu,\tau^{2}) \end{array} $$

where

10 $$\begin{array}{@{}rcl@{}} {\begin{aligned} p(y|\mu,\tau^{2};\hat{\lambda},\hat{\alpha})=\prod_{i=1}^{k}\left [\frac{1}{\sqrt{2\pi}(\tau^{2}+\phi_{i}^{2}(\hat{\lambda},\hat{\alpha}))^{1/2}}\right.\\ \left.\exp\left \{-\frac{(y_{i}(\hat{\lambda},\hat{\alpha})-\mu)^{2}}{2(\tau^{2}+\phi_{i}^{2}(\hat{\lambda},\hat{\alpha}))}\right \}\right ]. \end{aligned}} \end{array} $$

We assume the vague priors for *μ* and *τ* ^2^ in the same way as (2); i.e. *μ*∼*N*(0,10000) and *τ*∼*U*(0,*b*), where *b* is a constant value given by practitioners. It is straightforward to draw samples from the posterior distribution (10) by MCMC. The source code for conducting meta-analyses with the proposed model (7) is shown in Additional file 1, which includes the step of finding the maximum likelihood estimates of *λ* and *α*.

#### Interpretation of results

##### A median overall treatment effect

We first define a true effect of the untransformed variable *y*_*i*_ as

11 $$\begin{array}{@{}rcl@{}} \theta^{\ast}_{i}\equiv \left\{ \begin{array}{ll} \left\{\lambda\dot{g}(\alpha)^{\lambda-1}\mu_{i}+1\right\}^{1/\lambda}-\alpha, & \lambda \neq 0 \\ \exp\left\{{\frac{\mu_{i}}{\dot{g}(\alpha)}}\right\}-\alpha, & \lambda = 0 \\ \end{array} \right. \end{array} $$

which is derived by back-transforming the *μ*_*i*_. Since we are interested in estimating an overall effect in original scale before transformation (i.e. a centre of the distribution of $\theta _{i}^{\ast }$, not of *μ*_*i*_), it is useful to consider statistical measures induced from the distribution of $\theta _{i}^{\ast }$. Note that *μ*_*i*_∼*N*(*μ*,*τ* ^2^), then the *p*th percentile of the distribution of *μ*_*i*_ is given by *μ*+*τ* *z*_*p*_, where *z*_*p*_ denotes the *p*th percentile of a standard normal distribution. Thus, substituting *μ*+*τ* *z*_*p*_ into (11), we obtain the *p*th percentile of the distribution of $\theta _{i}^{\ast }$ as

12 $$\begin{array}{@{}rcl@{}} \xi_{p}= \left\{ \begin{array}{ll} \left\{\lambda\dot{g}(\alpha)^{\lambda-1}(\mu+\tau z_{p})+1\right\}^{1/\lambda}-\alpha, & \lambda \neq 0 \\ \exp\left\{{\frac{\mu+\tau z_{p}}{\dot{g}(\alpha)}}\right\}-\alpha, & \lambda = 0 \\ \end{array} \right.. \end{array} $$

And also, the median of the distribution of $\theta _{i}^{\ast }$ is given by

13 $$\begin{array}{@{}rcl@{}} \xi_{50}= \left\{ \begin{array}{ll} \left\{\lambda\dot{g}(\alpha)^{\lambda-1}\mu+1\right\}^{1/\lambda}-\alpha, & \lambda \neq 0 \\ \exp\left\{{\frac{\mu}{\dot{g}(\alpha)}}\right\}-\alpha, & \lambda = 0 \\ \end{array} \right.. \end{array} $$

The median (13) can now be used for the inference of an overall (summary) treatment effect on the original scale. We recommend using the median as a representative of centre of skewed distributions, which is more robust than the mean against the skewness and the outliers on the observed treatment effect estimates.

##### Quantification of heterogeneity using the ratio of IQR squares

Under the normal random effects model (1), the between-study variance *τ* ^2^ and the *I* ^2^ can be used for quantifying the magnitude and the impact of the heterogeneity across studies, respectively. However, when considering skewed distributions, variance is not the most appropriate measure for describing the spread of the distributions. In general, the variance is defined as an expected value of the squared deviation from the mean, though in the skewed-data situation the data is no longer distributed symmetrically around the mean. Due to the skewness or the heavy-tailedness of the data, the variance may lead a wrongly large spread of the distribution. That is, under the proposed model (7), the variance of the distribution of $\theta _{i}^{\ast }$ does not provide appropriate information about the heterogeneity across studies. For this reason, we here use an interquartile range (IQR) instead of the variance, which is defined as the difference between 75th and 25th quantiles for the distribution of $\theta _{i}^{\ast }$; i.e. *ξ*_75_−*ξ*_25_ from (12). Against the skewness of the data, the IQR is known as a more robust measure of spread than the variance. Note that the IQR of a normal distribution is exactly equal to the product of its standard deviation and *z*_75_−*z*_25_. Therefore, if we observe normally distributed treatment effect estimates, a measure of

14 $$\begin{array}{@{}rcl@{}} \frac{\xi_{75}-\xi_{25}}{z_{75}-z_{25}} \end{array} $$

from the proposed model (7) would be close to the square root of between-study variance from the normal random effects model (1). For this comparability, we recommend using the measure of (14), which is known as normalised IQR, for quantifying the magnitude of the heterogeneity.

We also define a criteria for quantifying the impact of the heterogeneity for the skewed treatment effects. Note that $y_{i}(\lambda,\alpha)\sim N\left (\mu,\tau ^{2}+\phi _{i}^{2}(\lambda,\alpha)\right)$, then the *p*th percentile of the distribution of *y*_*i*_(*λ*,*α*) is given by $\mu +(\tau ^{2}+\phi _{i}^{2}(\lambda,\alpha))^{1/2}z_{p}$. Substituting a ‘typical’ within-study variance like (4) into the $\phi _{i}^{2}(\lambda,\alpha)$ and back-transforming the *p*th percentile into the original scale, we obtain the *p*th percentile of the distribution of *y*_*i*_ as

15 $$  \nu_{p}= \left\{\!\!\! \begin{aligned} &\left\{\lambda\dot{g}(\alpha)^{\lambda-1}(\mu\,+\,(\tau^{2}\,+\,d^{2})^{1/2}z_{p})\,+\,1\right \}^{1/\lambda}\,-\,\alpha,\quad \lambda \neq 0 \\ &\exp\left \{{\frac{\mu+(\tau^{2}+d^{2})^{1/2}z_{p}}{\dot{g}(\alpha)}}\right \}-\alpha, \qquad\qquad\quad\!\lambda = 0 \\ \end{aligned} \right.  $$

where

$$\begin{array}{@{}rcl@{}} d^{2}=\frac{(k-1)\sum_{i=1}^{k} 1/\phi_{i}^{2}(\lambda,\alpha)}{\left(\sum_{i=1}^{k} 1/\phi_{i}^{2}(\lambda,\alpha)\right)^{2}-\sum_{i=1}^{k} \left(1/\phi_{i}^{2}(\lambda,\alpha)\right)^{2}} \end{array} $$

denotes the ‘typical’ within-study variance of the Box-Cox transformed variables. Obviously from the definition by (3), the *I* ^2^ has an aspect of the proportion of the between-study variation that is due to the heterogeneity across studies (variance of $\theta _{i}^{\ast }$) to the total variation in the treatment effect estimates (total variance of *y*_*i*_). In the similar concept, we now consider using a ratio of IQR squares alternative to the *I* ^2^, which is defined as

16 $$  \frac{(\text{IQR}\ \text{of~the~distribution~of} ~\theta_{i}^{\ast})^{2}}{(\text{IQR}\ \text{of~the}\ \text{distribution}\ \text{of}\ y_{i})^{2}}=\frac{(\xi_{75}-\xi_{25})^{2}}{(\nu_{75}-\nu_{25})^{2}}.  $$

The ratio of IQR squares would be comparable with the *I* ^2^ when the treatment effect estimates are normally distributed, because of the comparability between the IQR and the between-study variance.

##### Prediction interval

Under the proposed model (7), we first consider a predictive distribution of the Box-Cox transformed treatment effect which is given by

$$\begin{array}{@{}rcl@{}} p(\mu_{\text{new}}|y;\lambda,\alpha)=\int\!\!\!\int p(\mu_{\text{new}}|\mu,\tau^{2})p(\mu,\tau^{2}|y;\lambda,\alpha)d\mu d\tau^{2}. \end{array} $$

As described in the previous section, the sampling from *p*(*μ*_new_|*y*;*λ*,*α*) can be achieved by first drawing samples of parameters from the posterior distribution *p*(*μ*,*τ* ^2^|*y*;*λ*,*α*) and then drawing samples from *p*(*μ*_new_|*μ*,*τ* ^2^) with fixed parameters obtained in the previous step. In the second step, the drawing is performed by *μ*_new_∼*N*(*μ*,*τ* ^2^). Then, we obtain the samples from the predictive distribution of the treatment effect by back-transforming the samples of *μ*_new_ as

17 $$\begin{array}{@{}rcl@{}} \theta^{\ast}_{\text{new}}= \left\{ \begin{array}{ll} \left\{\lambda\dot{g}(\alpha)^{\lambda-1}\mu_{\text{new}}+1\right\}^{1/\lambda}-\alpha, & \lambda \neq 0 \\ \exp\left\{{\frac{\mu_{\text{new}}}{\dot{g}(\alpha)}}\right\}-\alpha, & \lambda = 0 \\ \end{array} \right.. \end{array} $$

A (100−*q*) percent prediction interval can be obtained by taking (*q*/2)th and (100−*q*/2)th quantiles of samples drawn from the predictive distribution (17). Again, note that this is just one option for obtaining the 95 percent prediction interval as mentioned in the previous section.

#### Another transformation for dealing with the negative skewness

As described in the previous section, the Box-Cox transformation (6) requires the condition that *y*_*i*_+*α* must be a positive value for *i*=1,…,*k*, which can cause difficulty in estimating the model parameters. This may also be more problematic when the treatment effect estimates have negative skewness, because the shift parameter is subject to inflation in such situation. In order to avoid the negative skewness on the treatment effect estimates, we here consider another transformation using a sign inversion. The transformation with the sign inversion described below will be applied only when the observed treatment effect estimates are negatively skewed.

We first distinguish which direction the skewness is in on the treatment effect estimates. A sample skewness with inverse-variance weightings defined as

18 $$\begin{array}{@{}rcl@{}} \frac{\sum_{i=1}^{k}\left.\left ({\frac{y_{i}-\bar{y}_{w}}{s_{w}}}\right)^{3}\right/\sigma_{i}^{2}}{\sum_{i=1}^{k} 1/\sigma_{i}^{2}} \end{array} $$

can be used for this, where

$$\begin{array}{@{}rcl@{}} \bar{y}_{w}=\frac{\sum_{i=1}^{k} y_{i}/\sigma_{i}^{2}}{\sum_{i=1}^{k} 1/\sigma_{i}^{2}},\quad s_{w}^{2}=\frac{\sum_{i=1}^{k} (y_{i}-\bar{y}_{w})^{2}/\sigma_{i}^{2}}{\sum_{i=1}^{k} 1/\sigma_{i}^{2}}. \end{array} $$

If the weighted sample skewness (18) take a negative value, we invert the sign of the treatment effect estimates (i.e. multiply the estimates by −1) and then apply the Box-Cox transformation to the inverted estimates. That is, we use the following transformation for the negatively skewed data:

19 $$\begin{array}{@{}rcl@{}} y_{i}(\lambda,\alpha)= \left\{ \begin{array}{ll} {\frac{(-y_{i}+\alpha)^{\lambda}-1}{\lambda\dot{h}(\alpha)^{\lambda-1}}}, & \lambda \neq 0 \\ \log(-y_{i}+\alpha)\dot{h}(\alpha), & \lambda = 0 \\ \end{array} \right. \end{array} $$

where $\dot {h}(\alpha)$ is now a geometric mean of −*y*_*i*_+*α* for *i*=1,…,*k*. For each study, the same within-study variances can be assigned to the inverted treatment effect estimates.

The random effects model (7) with the transformation (19) is applied in the same manner as the implementing procedures described in the previous section. And also, instead of (11), the true effect of the untransformed variable *y*_*i*_ is now defined as

$$\begin{array}{@{}rcl@{}} \theta^{\ast}_{i}\equiv \left\{ \begin{array}{ll} -\left\{\lambda\dot{h}(\alpha)^{\lambda-1}\mu_{i}+1\right\}^{1/\lambda}+\alpha, & \lambda \neq 0 \\ -\exp\left\{{\frac{\mu_{i}}{\dot{h}(\alpha)}}\right\}+\alpha, & \lambda = 0 \\ \end{array} \right.. \end{array} $$

Then, instead of (12) and (15), we have the *p*th percentiles of the distribution of $\theta _{i}^{\ast }$ and *y*_*i*_ as follows:

$$\begin{array}{@{}rcl@{}} \xi_{p}= \left\{ \begin{array}{ll} -\left\{\lambda\dot{h}(\alpha)^{\lambda-1}(\mu+\tau z_{p})+1\right\}^{1/\lambda}+\alpha, & \lambda \neq 0 \\ -\exp\left\{{\frac{\mu+\tau z_{p}}{\dot{h}(\alpha)}}\right\}+\alpha, & \lambda = 0 \\ \end{array} \right. \end{array} $$

and

$$ \nu_{p}\,=\,\left\{\!\!\! \begin{aligned} &-\left\{\lambda\dot{h}(\alpha)^{\lambda-1}(\mu\,+\,(\tau^{2}\,+\,d^{2})^{1/2}z_{p})\,+\,1\!\right\}^{1/\lambda}\!+\alpha,\,\,\, \lambda \neq 0\\ &-\exp\!\left\{{\frac{\mu+(\tau^{2}+d^{2})^{1/2}z_{p}}{\dot{h}(\alpha)}}\right \}+\alpha,\qquad\qquad\quad\!\! \lambda = 0 \\ \end{aligned} \right. $$

which can be used for estimating the overall median effect and the ratio of IQR squares. The prediction interval is also obtained by the same procedure described in the previous section, except for the step of back-transforming the samples of *μ*_new_. Instead of (17), we here use

$$\begin{array}{@{}rcl@{}} \theta^{\ast}_{\text{new}}= \left\{ \begin{array}{ll} -\left\{\lambda\dot{h}(\alpha)^{\lambda-1}\mu_{\text{new}}+1\right\}^{1/\lambda}+\alpha, & \lambda \neq 0 \\ -\exp\left\{{\frac{\mu_{\text{new}}}{\dot{h}(\alpha)}}\right\}+\alpha, & \lambda = 0 \\ \end{array} \right. \end{array} $$

for obtaining samples from the predictive distribution.

#### Implementation of our proposed model

We here summarise an implementation procedure of our proposed model using the Box-Cox transformation with the sign inversion for negatively skewed data:

1. Calculate the weighted sample skewness (18).
2. If the weighted sample skewness calculated in Step 1 takes a negative value, invert the sign of observed treatment effect estimates and then move to Step 3; otherwise just move to Step 3.
3. Calculate the maximum likelihood estimates of the transformation (*λ*) and the shift (*α*) parameter using the log-likelihood function (9).
4. Perform the Bayesian estimation (MCMC sampling) for the other parameters given the maximum likelihood estimates of the transformation and the shift parameter.
5. Calculate the measures of interest (overall median, normalised IQR and ratio of IQR squares) using the MCMC samples obtained in Step 4.
6. Draw samples from the predictive distribution using the MCMC samples obtained in Step 4, and calculate the prediction interval.

Steps 1 and 2 are needed only when applying the sign inversion. Without the sign inversion, we will begin the procedure from Step 3.

## Results

### Simulation study

We conducted a simulation study to examine the comparative performance of the standard normal random effects model (1) and the proposed model (7). Since the proposed model allows the presence of skewness on the treatment effect estimates, we supposed some situations where the true treatment effects had a skewed distribution, and compared results from the two models in terms of the estimation of overall effect and the quantification of heterogeneity. We also supposed another situation where the treatment effect estimates were normally distributed. In such situation, the two models are expected to provide similar results.

#### Design

Table 1 shows an overview of the simulation study. Under several scenarios of random effects distributions, we considered simulating 10,000 meta-analyses of *k* studies, where the number of studies was fixed in each simulation with *k*∈{5,10,20,40}. A treatment effect estimate *y*_*i*_ and a within-study variance $\sigma _{i}^{2}$ for the *i*th study (*i*=1,…,*k*) were randomly generated with the procedures of Steps 1-6 in Table 1. We below describe each step in detail.

**Table 1:** Overview of the simulation study

Step 1	Choose a random effects distribution *f*(*ψ*) from candidates including normal distributions, skew-normal distributions, shifted exponential distributions and shifted log-normal distributions, where *ψ* represents a true parameter vector of the random effects distribution.
Step 2	Choose the number of studies (*k*), mean of the distribution for the within-study variance (*σ* ^2^) and true parameters of the random effects distribution (*ψ*).
Step 3	Draw a within-study variance of the treatment effect estimate for the *i*th study (*i*=1,…,*k*); $\tilde {\sigma }_{i}^{2}\sim N(\sigma ^{2},0.040)$ conditioned on $0.010<\tilde {\sigma }_{i}^{2}<(2\sigma ^{2}-0.010)$.
Step 4	Draw a sampling error of the treatment effect estimate for the *i*th study (*i*=1,…,*k*); $\tilde {\epsilon }_{i}\sim N(0,\tilde {\sigma }_{i}^{2})$, where $\tilde {\sigma }_{i}^{2}$ is obtained in Step 3.
Step 5	Draw a true treatment effect for the *i*th study (*i*=1,…,*k*); $\tilde {\theta }_{i}\sim f(\psi)$, where *f*(*ψ*) is the specified random effects distribution with the true parameter *ψ*.
Step 6	Obtain a treatment effect estimate for the *i*th study (*i*=1,…,*k*); $\tilde {y}_{i}=\tilde {\theta }_{i}+\tilde {\epsilon }_{i}$, where $\tilde {\epsilon }_{i}$ and $\tilde {\theta }_{i}$ are obtained in step 4 and step 5 respectively.
Step 7	Using $\tilde {y}_{i}$ and $\tilde {\sigma }_{i}^{2}$ for *i*=1,…,*k*, fit the normal random effects model (1) and the proposed model (7) separately.
Step 8	Obtain a posterior median and a 95 percent credible interval of the overall mean from the normal random effects model (1), and those of the overall median from the proposed model (7). Check whether their credible intervals contain the true overall median of 0.000.
Step 9	Obtain a posterior median and a 95 percent credible interval of the *I* ^2^ from the normal random effects model (1), and those of the ratio of IQR squares from the proposed model (7). Check whether their credible intervals contain the true ratio of IQR squares given by one of either (20.0%, 40.0%, 80.0%).
Step 10	Repeat Steps 1 to Step 9 10,000 times.
Step 11	Using the posterior medians of the overall mean or the overall median obtained in Step 8, compute a bias and a root mean square error around the true overall median of 0.000.
Step 12	Obtain a coverage probability of the overall mean or the overall median by computing the proportion of the time that the 95 percent credible intervals contained the true overall median of 0.000.
Step 13	Using the posterior medians of the *I* ^2^ or the ratio of IQR squares obtained in Step 9, compute a bias and a root mean square error around the true ratio of IQR squares given by one of either (20.0%, 40.0%, 80.0%).
Step 14	Obtain a coverage probability of the *I* ^2^ or the ratio of IQR squares by computing the proportion of the time that the 95 percent credible intervals contained the true ratio of IQR squares given by one of either (20.0%, 40.0%, 80.0%).

In Step 1, a random effects distribution was chosen from candidates. We considered a variety of random effects distributions (normal distribution, skew-normal distribution [40, 41], shifted exponential distribution and shifted log-normal distribution) which a true treatment effect *θ*_*i*_ for the *i*th study was drawn from. The normal distributions were chosen for examining how the proposed model worked in the case of symmetrically distributed data that could be precisely fit by the normal random effects model. The skew-normal distributions were chosen for imitating situations with moderate to large skewness in a positive and a negative directions. The shifted exponential and the shifted log-normal distributions were chosen for imitating situation with heavy-tailed data as well as positive skewness. True parameters in the random effects distributions were specified so that the median of the distribution became equal to zero, and the normalised IQR square of the distribution became one of either (0.025, 0.067, 0.400). The setting of zero overall median means a null hypothesis of no treatment effect. The scenario of the true normalised IQR square is equivalent to setting the true ratio of the IQR squares as (20.0%, 40.0%, 80.0%) which are obtained by plugging in the true normalised IQR squares under the ‘typical’ within-study variance of 0.100, such as 0.200=0.025/(0.025+0.100). Table 2 shows the values of true parameters included in each random effects distribution. And also, the random effects distributions are graphically illustrated for each scenario in Additional file 2: Figure S1, Figure S2, Figure S3, Figure S4, Figure S5, Figure S6 and Figure S7 show density functions of the random effects distribution for the scenarios 1-3, 4-6, 7-9, 10-12, 13-15, 16-18 and 19-21, respectively.

**Table 2:** Scenarios of random effects distributions and their true parameters

Random effects				Normalised	Ratio of
distribution	Scenario	True parameter	Median	IQR	IQR squares
Scenario 1-3: Normal distribution (N)					
*N*(mean,variance)	1	*N*(0,0.1581^2^)	0.000	0.025	20.0%
	2	*N*(0,0.2582^2^)	0.000	0.067	40.0%
	3	*N*(0,0.6325^2^)	0.000	0.400	80.0%
Scenario 4-6: Skew-normal distribution with moderate positive skewness (pSN1)					
*SN*(location,scale,slant)	4	*S* *N*(−0.1724,0.2547,5)	0.000	0.025	20.0%
	5	*S* *N*(−0.2812,0.4159,5)	0.000	0.067	40.0%
	6	*S* *N*(−0.6883,1.0198,5)	0.000	0.400	80.0%
Scenario 7-9: Skew-normal distribution with large positive skewness (pSN2)					
*SN*(location,scale,slant)	7	*S* *N*(−0.1734,0.2557,20)	0.000	0.025	20.0%
	8	*S* *N*(−0.2829,0.4192,20)	0.000	0.067	40.0%
	9	*S* *N*(−0.6923,1.0258,20)	0.000	0.400	80.0%
Scenario 10-12: Skew-normal distribution with moderate negative skewness (nSN1)					
*SN*(location,scale,slant)	10	*S* *N*(0.1715,0.2546,−5)	0.000	0.025	20.0%
	11	*S* *N*(0.2803,0.4154,−5)	0.000	0.067	40.0%
	12	*S* *N*(0.6874,1.0195,−5)	0.000	0.400	80.0%
Scenario 13-15: Skew-normal distribution with large negative skewness (nSN2)					
*SN*(location,scale,slant)	13	*S* *N*(0.1725,0.2556,−20)	0.000	0.025	20.0%
	14	*S* *N*(0.2820,0.4191,−20)	0.000	0.067	40.0%
	15	*S* *N*(0.6914,1.0255,−20)	0.000	0.400	80.0%
Scenario 16-18: Shifted exponential distribution (EXP)					
*EXP*(rate,shift)	16	*E* *X* *P*(5.1507,−0.1348)	0.000	0.025	20.0%
	17	*E* *X* *P*(3.1542,−0.2202)	0.000	0.067	40.0%
	18	*E* *X* *P*(1.2877,−0.5377)	0.000	0.400	80.0%
Scenario 19-21: Shifted log-normal distribution (LN)					
*LN*(mean,variance,shift)	19	*L* *N*(0,0.1578^2^,−1)	0.000	0.025	20.0%
	20	*L* *N*(0,0.2569^2^,−1)	0.000	0.067	40.0%
	21	*L* *N*(0,0.6147^2^,−1)	0.000	0.400	80.0%

In Step 2, we set the number of studies, mean of the distribution for the within study variance and true parameters of the random effects distribution. In Steps 3-6, we obtained treatment effect estimates for each study. In particular, the within-study variance $\sigma _{i}^{2}$ was drawn from a normal distribution with mean *σ* ^2^ and variance 0.040 conditioned on $0.010<\sigma _{i}^{2}<(2\sigma ^{2}-0.010)$. The mean of the normal distribution, *σ* ^2^, was chosen so that the ‘typical’ within-study variance (4), which depended on the number of studies involved in the meta-analysis, became 0.100 on average. We set the value of *σ* ^2^ to either 0.1089, 0.1122, 0.1147, 0.1158 in each simulation with *k*=5,10,20,40, respectively.

In Step 7, using the generated meta-analysis data, we fit the normal random effects model (1) and the proposed model (7) separately. In the proposed model, we also applied the transformation with the sign inversion for the negatively skewed data. Note that the transformation with the sign inversion is applied only when the observed treatment effect estimates are negatively skewed. And then, in Steps 8-9, we computed the posterior medians and the 95 percent credible intervals of: the overall mean and the *I* ^2^ from the normal random effects model (1), the overall median and the ratio of IQR squares from the proposed model (7).

In Steps 11-14, we calculated the following quantities for comparing the two models (normal random effects model/proposed model):

- Bias around the true overall median: (mean of the posterior medians of the overall mean/the overall median) −(true overall median of 0.000)
- Root mean square error (RMSE) around the true overall median: ((standard deviation of the posterior medians of the overall mean/the overall median) ^2^+(bias around the true overall median)^2^) ^1/2^
- Coverage probability of the true overall median (%): the proportion of the time that the 95 percent credible intervals of the overall mean/the overall median contained the true overall median of 0.000
- Bias around the true ratio of IQR squares: (mean of the posterior medians of the *I* ^2^/the ratio of IQR squares) −(true ratio of IQR squares given by one of either (20.0%, 40.0%, 80.0%))
- RMSE around the true ratio of IQR squares: ((mean of the posterior medians of the *I* ^2^/the ratio of IQR squares) ^2^+(bias around the true ratio of IQR squares)^2^) ^1/2^
- Coverage probability of the true ratio of IQR squares (%): the proportion of the time that the 95 percent credible intervals of the *I* ^2^/the ratio of IQR squares contained the true ratio of IQR squares given by one of either (20.0%, 40.0%, 80.0%)

We notice that using the terms of bias, RMSE and coverage probability for the results from the normal random effects model is not necessarily correct. This is because the normal random effects model provided the results of overall mean and *I* ^2^, which were not the targeted true values (or the reference values). However, in this article, the overall median and the ratio of IQR squares are highly recommended for representing the overall effect and quantifying the heterogeneity in the meta-analysis of skewed data, respectively. Then, the above quantities are useful for assessing how the findings under the two models can be different from the recommended inferential measures in skewed-data situations.

#### Estimation

Before estimation of model (7) for a particular simulated dataset, the grid search procedure was performed for estimating *λ* and *α* for the dataset. The candidate values of *λ* were specified in a range of −3.00≤*λ*≤6.00 with a step size of 0.01. We considered constituting a subset of *α* as the minimum values of {(*y*_*i*_+*α*):*i*=1,…,*k*}; i.e. *α* ^∗^=*α*+ min{*y*_*i*_:*i*=1,…,*k*}. The candidate values of *α* ^∗^ were specified in a range of 0.01≤*α* ^∗^≤2.01 with a step size of 0.10.

We used the normal and the uniform prior for the mean and the variance parameter respectively, as described in the previous section. The upper limit of the uniform prior distribution on *τ* was given by *b*=10 for each model. For the Bayesian estimation of model (1) and (7), the iterative process of the MCMC algorithm produced three chains each with 20,000 samples of parameters. We discarded the first 2,000 samples (so-called burn-in samples) in order to prevent dependence on the starting values. And also, we took a sample at only every 2nd iteration (thinning) in order to avoid autocorrelation between the samples taken. Therefore in total, 24,000 samples of parameters were drawn. We graphically checked the convergence of MCMC sampling using first 5 simulations for each scenario, with no diagnostic methods.

#### Results

**Fig. 2:**
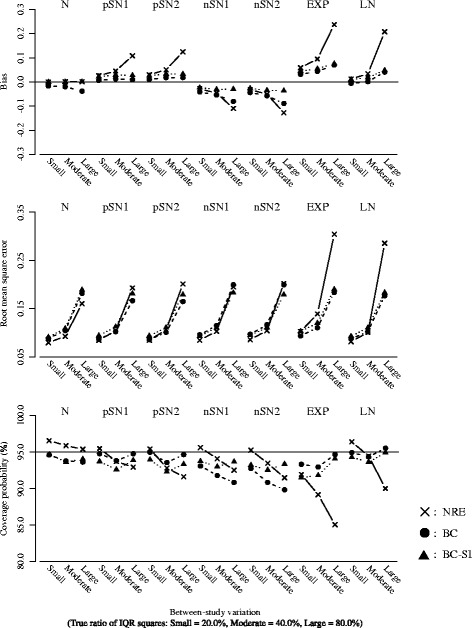
Bias, RMSE and coverage probability of the overall mean or the overall median for the scenario of the number of studies *k*=20. The overall mean from the normal random effects model (*cross/solid line*), and those of the overall median from the proposed model (*black circle/broken line*: Box-Cox transformation, *black triangle/dotted line*: Box-Cox transformation with the sign inversion)

**Fig. 3:**
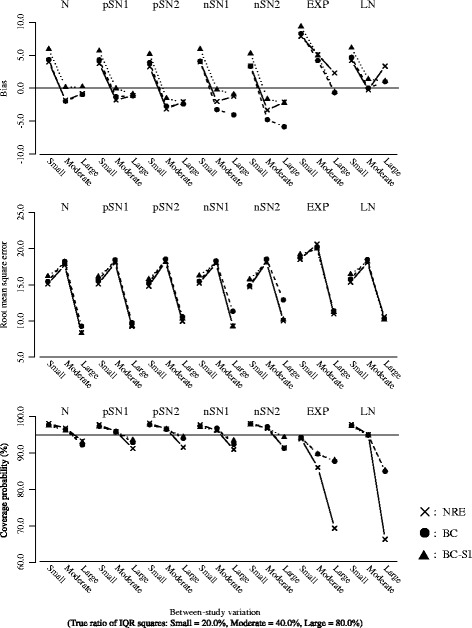
Bias, RMSE and coverage probability of the *I* ^2^ or the ratio of IQR squares for the scenario of the number of studies *k*=20. The *I* ^2^ from the normal random effects model (*cross/solid line*), and those of the ratio of IQR squares from the proposed model (*black circle/broken line*: Box-Cox transformation, *black triangle/dotted line*: Box-Cox transformation with the sign inversion)

**Fig. 4:**
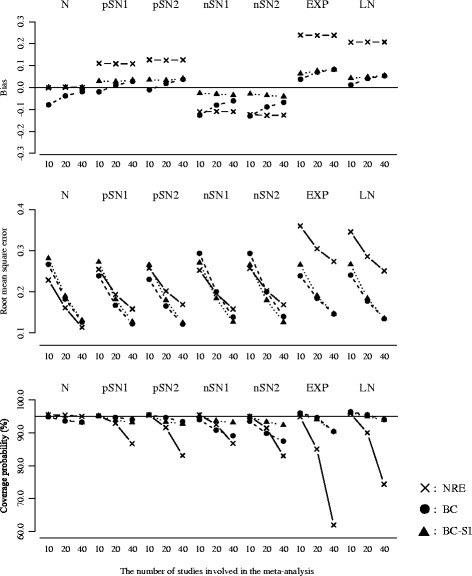
Bias, RMSE and coverage probability of the overall mean or the overall median for the scenario of true ratio of IQR squares = 80.0% (large between-study variation). The overall mean from the normal random effects model (*cross/solid line*), and those of the overall median from the proposed model (*black circle/broken line*: Box-Cox transformation, *black triangle/dotted line*: Box-Cox transformation with the sign inversion)

**Fig. 5:**
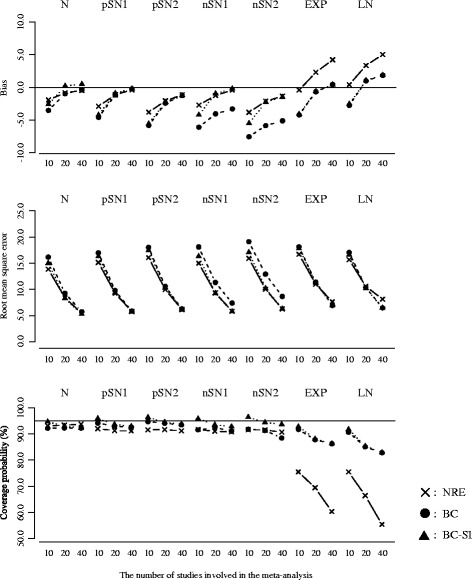
Bias, RMSE and coverage probability of the *I* ^2^ or the ratio of IQR squares for the scenario of true ratio of IQR squares = 80.0% (large between-study variation). The *I* ^2^ from the normal random effects model (*cross/solid line*), and those of the ratio of IQR squares from the proposed model (*black circle/broken line*: Box-Cox transformation, *black triangle/dotted line*: Box-Cox transformation with the sign inversion)

Additional file 2: Table S1, Table S2, Table S3 and Table S4 show results from the two models, for each scenario of the number of studies *k* = 5, 10, 20 and 40, respectively. And also, Additional file 2: Table S5 and Table S6 show summary statistics of estimates for the transformation (*λ*) and the shift (*α*) parameter, for the scenario of the number of studies *k*=40. Note that the summary statistics were calculated using 10,000 estimates of the parameters for each scenario of random effects distribution. To make clear the differences between the two models, we depicted the bias, the RMSE and the coverage probability in the following figures:

- Figure 2 plots the results for the overall mean or the overall median, with the between-study variation (the true ratio of IQR squares: Small = 20.0%, Moderate = 40.0%, Large = 80.0%) on the horizontal axis. The number of studies was fixed as *k* = 20.
- Figure 3 plots the results for the *I* ^2^ or the ratio of IQR squares, with the between-study variation (the true ratio of IQR squares: Small = 20.0%, Moderate = 40.0%, Large = 80.0%) on the horizontal axis. The number of studies was fixed as *k* = 20.
- Figure 4 plots the results for overall mean or the overall median, with the number of studies (*k* = 10, 20 and 40) on the horizontal axis. The true ratio of IQR squares was fixed as 80.0% (i.e. the scenario of large between-study variation).
- Figure 5 plots the results for the *I* ^2^ or the ratio of IQR squares, with the number of studies (*k* = 10, 20 and 40) on the horizontal axis. The true ratio of IQR squares was fixed as 80.0% (i.e. the scenario of large between-study variation).

The nominal level of coverage probability is 95 percent. All the scenarios of the random effects distributions are displayed in the same panels in order of the normal (N), the skew-normal with moderate positive skewness (pSN1), the skew-normal with large positive skewness (pSN2), the skew-normal with moderate negative skewness (nSN1), the skew-normal with large negative skewness (nSN2), the shifted exponential (EXP) and the shifted log-normal (LN) from left to right. And also, in each figure, (i) cross marks and solid lines represent the normal random effects model, (ii) black circle marks and broken lines represent the proposed model using Box-Cox transformation (6), (iii) black triangle marks and dotted lines represent the proposed model using Box-Cox transformation with the sign inversion (19) for the negatively skewed data. We below refer to the normal random effects model, the proposed model without and with the sign inversion as NRE, BC and BC-SI respectively.

##### Overall treatment effect

When the normal distributions were assumed as the true random effects distribution, the NRE and the BC-SI provided unbiased estimations of the overall effect, regardless of the scenarios of the between-study variation and the number of studies. The overall median from the BC was subject to a negative bias in the scenario of the large between-study variation and the small number of studies, though this bias decreased as the number of studies increased. The NRE, the BC and the BC-SI provided similar RMSEs, except for the scenario of the small number of studies where the RMSEs from the NRE were smaller than those from the BC and the BC-SI. The coverage probabilities from the NRE were slightly larger than those from the BC and the BC-SI in all the scenarios, but all these coverage probabilities were close to the nominal level of 95 percent.

When the skew-normal distributions were assumed as the true random effects distribution, the overall means from the NRE were pulled in the direction of skewness and substantially different from the true zero overall median, especially in the scenarios of the large between-study variation. In the scenarios of the positive skewness (pSN1 and pSN2), the biases of the overall mean from the NRE increased positively; conversely in the scenarios of negative skewness (nSN1 and nSN2), those increased negatively. The degree of bias was larger in the scenario of the large skewness (pSN2 and nSN2). And also, regarding the overall means from the NRE in the scenarios of the large between-study variation and the large number of studies, the RMSEs were inflated and the coverage probabilities were substantially below the nominal level of 95 percent. On the other hand, the overall medians from the BC and the BC-SI had the smaller biases regardless of the scenarios of the between-study variation and the number of studies. In the scenario of the negative skewness (nSN1 and nSN2) and the large between-study variation, the BC was subject to a negative bias, though this bias decreased as the number of studies increased. The BC and the BC-SI provided quite similar RMSEs and coverage probabilities in the scenarios of the positive skewness (pSN1 and pSN2); while, in the scenarios of the negative skewness (nSN1 and nSN2) and the large between-study variation, the coverage probabilities from the BC were below the nominal level of 95 percent. This indicates that the BC could have difficulty in dealing with the negatively skewed data as expected, but the BC-SI performs well.

When the shifted exponential and the shifted log-normal distributions were assumed as the true random effects distribution, the overall means from the NRE were substantially different from the true zero overall median, especially in the scenarios of the large between-study variation. And also, in such situation, the RMSEs were seriously inflated and the coverage probabilities were below the nominal level of 95 percent, which became more noticeable as the number of studies increased. This would be because the scenarios including larger number of studies tended to generate more heavy-tailed data. On the other hand, the overall medians from the BC and the BC-SI were similar and had much smaller biases in comparison with the overall means from the NRE. The BC and the BC-SI also provided similar results of the RMSE and the coverage probability, which were much better than those from the NRE especially in the scenarios of the large between-study variation and the large number of studies.

In summary, we found that the overall mean from the NRE could be substantially influenced by the skewness on the random effects distribution. Taking into account that the overall mean from the NRE was pulled in the direction of skewness and had the lower coverage probability, the NRE might therefore produce overall effect estimates that do not reflect the median treatment effect if the overall distribution of treatment effect estimates is skewed or heavy-tailed. Moreover, it was indicated that the sign inversion in the Box-Cox transformation could be an effective way for precisely estimating the overall median of the negatively skewed treatment effect estimates.

##### Quantification of heterogeneity

When the normal distributions were assumed as the true random effects distribution, the NRE, the BC and the BC-SI provided similar results of the bias, the RMSE and the coverage probability, regardless of the scenarios of the between-study variation and the number of studies.

When the skew-normal distributions were assumed as the true random effects distribution, the NRE, the BC and the BC-SI provided similar results in almost all of the scenarios. In the scenarios of the large between-study variation, the coverage probabilities of *I* ^2^ from the NRE were slightly lower than those of the ratios of IQR squares from the BC-SI. In the scenarios of the negative skewness (nSN1 and nSN2), the ratios of IQR squares from the BC were subject to negative biases and had the larger RMSEs in comparison with the NRE and BC-SI. This again indicates that the BC could have difficulty in dealing with the negatively skewed data.

When the shifted exponential and the shifted log-normal distributions were assumed as the true random effects distribution, the *I* ^2^ values from the NRE were larger than the ratios of IQR squares from the BC and the BC-SI in the scenarios of the large between-study variation. The RMSEs from the NRE, the BC and the BC-SI were quite similar, though the coverage probabilities of *I* ^2^ from the NRE were seriously below the nominal level of 95 percent in the scenarios of the large between-study variation, compared with those of the ratios of IQR squares from the BC and the BC-SI. This became more noticeable as the number of studies increased, which would be again because the scenarios including larger number of studies tended to generate more heavy-tailed data. The BC and the BC-SI provided quite similar results of the bias, the RMSE and the coverage probability, regardless of the scenarios of the between-study variation and the number of studies.

In summary, we found that the *I* ^2^ from the NRE was influenced by the skewness on the random effects distribution. In particular, the heavy-tailed data seriously affected the estimation of *I* ^2^ in the NRE. Moreover, it was again indicated that the sign inversion in the Box-Cox transformation could be an effective way for precisely estimating the ratios of IQR squares of the negatively skewed treatment effect estimates.

##### Performance when the number of studies is small

Additional file 2: Table S1 shows results for the scenario of the number of studies *k*=5. In regard to the estimation of the overall treatment effect, having small number of studies had a limited influence on bias. Indeed, the biases on the overall median from the BC and the BC-SI as well as the overall mean from the NRE were similar to those for the scenario of the number of studies *k*=10, except for the overall median from the BC for the scenario of the negative skewness (nSN1 and nSN2) where the negative biases were increased. However, the coverage probabilities from the BC and the BC-SI were below nominal level of 95 percent for almost all the scenarios. In particular, the BC-SI provided around 90 percent coverage probabilities for the scenarios of the small and the moderate between-study variation. In contract, the coverage probabilities from the NRE were substantially above the nominal level of 95 percent. These indicate an issue of meta-analysing the small number of studies. In regard to the quantification of heterogeneity, the NRE, the BC and the BC-SI were subject to large positive bias of the *I* ^2^ or the ratio of IQR squares, which inflated their RMSEs. From these findings, we conclude our proposed model is applicable even when the number of studies is 5, but may have difficulty in ensuring sufficient accuracy in estimation of the overall treatment effect and quantification of heterogeneity.

### Application

Consider now application to the examples described in the previous section. We applied the normal random effects model (1) and the proposed model (7) to each example, and estimated the posterior distributions of parameters of interest in each model. The transformation with the sign inversion was also applied to example 2 (the weighted sample skewnesses were 2.123 and −1.847 in example 1 and 2 respectively). Note that the transformation with the sign inversion is applied only when the observed treatment effect estimates are negatively skewed.

#### Estimation

Before estimation of model (7) for each example, the grid search procedure was performed for estimating *λ* and *α*. The candidate values of *λ* were specified in a range of −3.00≤*λ*≤6.00 with a step size of 0.01. We considered constituting a subset of *α* as the minimum values of {(*y*_*i*_+*α*):*i*=1,…,*k*}; i.e. *α* ^∗^=*α*+ min{*y*_*i*_:*i*=1,…,*k*}. The candidate values of *α* ^∗^ was specified in a range of 0.01≤*α* ^∗^≤2.01 with a step size of 0.10.

We used the normal and the uniform prior for the mean and the variance parameter respectively, as described in the previous section. The upper limit of the uniform prior distribution on *τ* was given by *b*=10 for each model. For the Bayesian estimation, the iterative process of the MCMC algorithm produced three chains each with 2,000,000 samples of parameters. We discarded the first 5000 samples (so-called burn-in samples) in order to prevent dependence on the starting values. And also, we took a sample at only every 5th iteration (thinning) in order to avoid autocorrelation between the samples taken. Therefore in total, 1,185,000 samples of parameters were drawn. We again graphically checked whether the burn-in samples were sufficient and that the MCMC chains converged, with no diagnostic methods.

#### Overall treatment effect and quantification of heterogeneity

Table 3 shows the posterior median and the 95 percent credible interval of: the overall mean and the square root of between-study variance from the NRE, the overall median and the normalised IQR from the BC and the BC-SI. In example 1, the posterior median of the overall mean from the NRE was noticeably larger than that of the overall median from the BC. In example 2, the posterior medians of the overall mean from the BC and the BC-SI were quite similar to each other, but noticeably larger than that of the overall mean from the NRE. Note that the observed treatment effect estimates in example 1 were subject to the positive skewness, in contrast we observed the negatively skewed treatment effect estimates in example 2; this causes the overall means from NRE to be forced toward the direction of skewness in each example.

**Table 3:** Posterior median and 95 percent credible interval of: overall mean and square root of between-study variance from the normal random effects model, overall median and normalised IQR from the proposed model

NRE		BC		BC-SI	
	Square root of				
	between-study		Normalised		Normalised
Overall mean	variance	Overall median	IQR^a^	Overall median	IQR^a^
Post. (s.d.)	Post. (s.d.)	Post. (s.d.)	Post. (s.d.)	Post. (s.d.)	Post. (s.d.)
(95% CI)	(95% CI)	(95% CI)	(95% CI)	(95% CI)	(95% CI)
Example 1: Teacher expectancy on pupil IQ					
0.083 (0.061)	0.146 (0.087)	0.030 (0.051)	0.084 (0.074)	n/a	n/a
(−0.021,0.222)	(0.011,0.344)	(−0.058,0.144)	(0.004,0.278)		
Example 2: Antidepressants for reducing pain in fibromyalgia syndrome					
−0.418 (0.067)	0.164 (0.097)	−0.369 (0.056)	0.094 (0.077)	−0.361 (0.057)	0.098 (0.081)
(−0.567,−0.298)	(0.013,0.384)	(−0.489,−0.267)	(0.005,0.291)	(−0.484,−0.259)	(0.005,0.306)

^a^Normalised IQR =(*ξ*_75_−*ξ*_25_)/(*z*_75_−*z*_25_)

Post.: posterior median, s.d.: standard deviation, CI: credible interval

NRE: normal random effects model, BC: proposed model using Box-Cox transformation

BC-SI: proposed model using Box-Cox transformation with the sign inversion for negatively skewed data

**Fig. 6:**
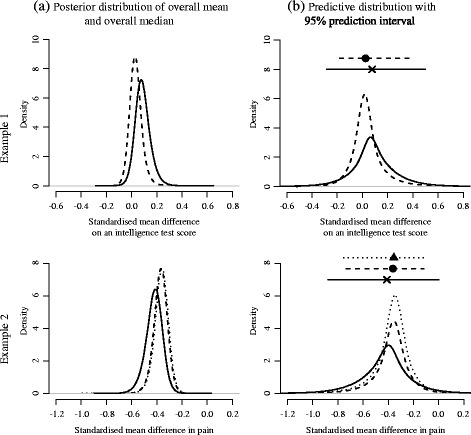
Posterior and predictive distribution. **a** Posterior distribution of the overall mean from the normal random effects model (*solid line*), and of the overall median from the proposed model (*broken line*: Box-Cox transformation, *dotted line*: Box-Cox transformation with the sign inversion), **b** Predictive distribution with 95 percent prediction interval from the normal random effect model (*solid line*), and those from the proposed model (*black circle/broken line*: Box-Cox transformation, *black triangle/dotted line*: Box-Cox transformation with the sign inversion)

In both examples, the 95 percent credible intervals of the overall median from the BC and the BC-SI were substantially narrower than those of the overall mean from the NRE, indicating the misspecification of the random effects distribution led to the inflation of the between-study variance in the NRE. Indeed, in both examples, we found larger posterior medians of the square root of between-study variance from the NRE in comparison with the normalised IQRs from the BC and the BC-SI. Figure 6a shows the posterior distributions of the overall mean from the NRE and those of the overall median from the BC and the BC-SI for each example. The overall medians had sharper peak of posterior densities than the overall mean in both examples.

Table 4 shows the posterior medians and the 95 percent credible intervals of the *I* ^2^ from the NRE, and the ratio of IQR squares from the BC and the BC-SI. In example 2, the results from the BC and the BC-SI were quite similar. The ratios of IQR squares from the BC and the BC-SI were substantially smaller than the *I* ^2^’s from the NRE in both examples. The NRE would conclude moderate heterogeneity for the meta-analyses of the examples; however, taking into account the inflation of the between-study variance from the NRE, the *I* ^2^’s are more likely to be overestimated. On the other hand, the BC and the BC-SI would conclude low heterogeneity for the same examples.

**Table 4:** Posterior median and 95 percent credible interval of: *I* ^2^ from the normal random effects model, ratio of IQR squares from the proposed model; 95 percent prediction intervals from each model

NRE		BC		BC-SI	
		Ratio of IQR		Ratio of IQR	
*I* ^2^ (%)	95%	squares^a^ (%)	95%	squares^a^ (%)	95%
Post. (s.d.)	prediction	Post. (s.d.)	prediction	Post. (s.d.)	prediction
(95% CI)	interval	(95% CI)	interval	(95% CI)	interval
Example 1: Teacher expectancy on pupil IQ					
44.9 (24.0)	(−0.284,0.500)	20.9 (21.9)	(−0.179,0.393)	n/a	n/a
(0.5,81.9)		(0.1,73.6)			
Example 2: Antidepressants for reducing pain in fibromyalgia syndrome					
39.1 (22.7)	(−0.879,−0.001)	17.3 (19.1)	(−0.732,−0.118)	18.4 (19.8)	(−0.753,−0.112)
(0.4,78.0)		(0.1,65.7)		(0.1,67.9)	

^a^Ratio of IQR squares =(*ξ*_75_−*ξ*_25_)^2^/(*ν*_75_−*ν*_25_)^2^

Post.: posterior median, s.d.: standard deviation, CI: credible interval

NRE: normal random effects model, BC: proposed model using Box-Cox transformation

BC-SI: proposed model using Box-Cox transformation with the sign inversion for negatively skewed data

#### Prediction interval and predictive probability

Table 4 also shows the 95 percent prediction intervals from the two models. In example 2, the results from the BC and the BC-SI were quite similar. In both examples, the prediction intervals from the BC and the BC-SI were substantially narrower than those from the NRE. This is likely due to the inflation of the between-study variance from the NRE. Especially in example 2, the BC and the BC-SI provided much stronger evidence of efficacy of the treatment, with the upper bound of the 95 percent prediction interval now much further below 0. Figure 6b shows the predictive distributions from the NRE, the BC and the BC-SI for each example. The 95 percent prediction intervals were also depicted on the same panel, where the cross, the black circle and the black triangle represent the medians of predictive distribution from the NRE, the BC and the BC-SI, respectively. We found the BC and the BC-SI provided skewed prediction intervals, which reflects the asymmetry detected and the asymmetric predictive distribution; whereas the NRE method gave symmetrical prediction intervals in both examples.

**Fig. 7:**
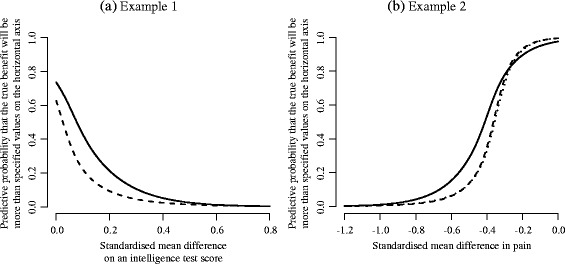
Predictive probability. The normal random effect model (*solid line*) and the proposed model (*broken line*: Box-Cox transformation, *dotted line*: Box-Cox transformation with the sign inversion)

We computed the predictive probability that the treatment is truly effective in a new study. Figure 7 shows the results from the NRE, the BC and the BC-SI. Note that the predictive probability is a kind of cumulative probability and is defined for each example as follows: *P*(*θ*_new_>*x*) or $P(\theta ^{\ast }_{\text {new}}>x)$ for example 1 (larger is more beneficial), and *P*(*θ*_new_<*x*) or $P(\theta ^{\ast }_{\text {new}}<x)$ for example 2 (smaller is more beneficial) where *x* is a specified value of treatment effect and is represented on the horizontal axis in Fig. 7. We below describe the details of computation and the results for each example:

##### Example 1.

Since a positive value indicates a higher mean score for the treatment group, we obtained the predictive probability of a beneficial treatment by counting the number of samples drawn from the predictive distribution which were larger than specified values on the horizontal axis. The probability curve from the NRE was located entirely over those from the BC. That is, the NRE predicted larger probabilities of the true effect being in favour of treatment than the BC. For instance, the probability of the true treatment effect being above 0.1 was 0.428 for the NRE but 0.221 for the BC.

##### Example 2.

Since a negative value indicates a benefit for the antidepressants, we obtained the predictive probability by counting the number of samples drawn from the predictive distribution which were smaller than specified values on the horizontal axis. The results from the BC and the BC-SI were quite similar. When the size of specified difference was small (e.g. from −0.3 to 0.0), the predictive probabilities from the NRE were slightly smaller than those from the BC and the BC-SI. In contrast, when the size of specified difference was large (e.g. from −0.8 to −0.4), the predictive probabilities from the NRE were larger than those from the BC and the BC-SI.

## Discussion

We proposed a new random effects model based on the Box-Cox transformation to deal with skewness in the overall distribution of the observed treatment effect estimate for meta-analysis. The simulation study shows that the proposed model has the potential to provide more appropriate inferences in the presence of skewness, especially in regard to the estimation of the overall treatment effect and the quantification of heterogeneity. The simulation study indicates that the normal random effects model gives an overall mean that is pulled in the direction of skewness, and is thus an inappropriate summary for representing the centre of skewed data. Similarly, *I* ^2^ from the normal random effects model can be inflated given skewed treatment effect estimates, because it overestimates the random effects variance. This also causes prediction intervals that are too wide.

Our proposed model substantially reduces these problems. It is flexible that the observed data determine the shape of distribution and thus the required Box-Cox transformation to ensure the normality of transformed treatment effect estimates. We suggest using the overall median effect to summarise the proposed meta-analysis model, back on the original scale of interest. The median is known as a more robust summary measure than the mean against the skewness and the outliers on the observed data. We also defined the ratio of IQR squares under the proposed model for quantifying the impact of heterogeneity in the meta-analysis. When considering the skewed data, the variance is no longer the best measure for describing the spread of the distribution. We recommend the (normalised) IQR of the true effects distribution as a measure for quantifying the extent of the heterogeneity. The ratio of IQR squares has an aspect of the proportion of the between-study variation that is due to the heterogeneity across studies to the total variation in the treatment effect estimates, which is the same concept as *I* ^2^ from the normal random effects model. In the simulation study, the ratio of IQR squares reduced the inflation of *I* ^2^ when the treatment effect estimates were skewed or heavy-tailed.

We note that our simulation assumes that sample sizes in each study are large enough for the central limit theorem to apply, such that (a) treatment effect estimates do have a normal distribution within studies, (b) the variance of the estimate is well-estimated (such that it can essentially be assumed known). Thus, situations with small studies are not considered, but this would be useful for further research.

The application to the two examples illustrated the two models could provide different conclusions for the summary effect and the amount of heterogeneity for the same meta-analysis data. In addition, given skewness the applications indicate the proposed model better predicts the treatment effect in a new study over the normal random effects model. The normal random effects model provided symmetric predictive distributions and its 95 percent prediction interval; on the other hand, the proposed model provided the skewed shape of predictive distributions and its asymmetric 95 percent prediction interval as expected. The difference in the shape of predictive distributions had a significant impact on the predictive probabilities that the treatment is effective in a new study. Another limitation is that, although our simulations covered a wide range of scenarios and were computationally intensive, other scenarios still need to be investigated. In particular, we did not consider when sample sizes within studies are small, and we only considered when it could be correctly assumed that study estimates were normally distributed and their variances were known. This allowed any asymmetry to be due to the random effects distribution, rather than the within-study distributions. Further research in situations of small trials and/or rare events would be welcome.

Note that the parameters included in our proposed model are estimated by two stages. We first get the point estimates of the transformation and the shift parameter (*λ* and *α*) using the profile likelihood function, and then estimate the other parameters (*μ* and *τ*) using the Bayesian approach conditioned on $\lambda =\hat {\lambda }$ and $\alpha =\hat {\alpha }$. Although a simultaneous estimation of all parameters (*λ*, *α*, *μ* and *τ*) within the framework of Bayesian approach is more straightforward, we have had a difficulty in the convergence of MCMC sampling for this. Therefore, we take a procedure of first finding a transformation to normalise the treatment effect estimates (i.e. the transformation and the shift parameter are dealt with as non-stochastics), and then making inferences conditioned on the maximum likelihood estimates of the transformation and the shift parameter.

However, there are some limitations of the proposed model and further research is required. For interpretation and presentation of the meta-analysis results, the Bayesian approach is used for estimating the model parameters. We are interested in functions of the estimated parameters rather than the estimated parameters themselves; i.e. (i) the overall median (13) which represents a summary treatment effect, (ii) the normalised IQR (14) which quantifies the magnitude of heterogeneity, (iii) the ratio of IQR squares (16) which quantifies the impact of heterogeneity. The Bayesian approach using the MCMC method is straightforward enough to estimate these measures with variability, because uncertainty (i.e. variance estimation) of the estimated measures can be obtained by using MCMC samples directly (e.g. mean, median, standard deviation, 2.5th and 97.5th quantiles of the MCMC samples), without additional distributional assumptions or asymptotic approximations. The 95 percent prediction interval is also computed in a simple manner by the Bayesian approach, which can be obtained by taking 2.5th and 97.5th quantiles of samples drawn from the predictive distribution (17). However, a frequentist approach may be another useful option in some situations. When considering the frequentist estimation for the proposed model, it is not straightforward to derive asymptotic distributions (and also the 95 percent confidence intervals) of the maximum likelihood estimators for the measures of interest. We expect a bootstrap method can be used for solving this issue. In addition, it is not necessarily clear how the choice of prior for the between-study variance parameter makes impact on the results from our proposed model. The uniform prior on the standard deviation scale has been known as a reasonable non-informative prior for the conventional normal random effects model, though this may not be the case for our proposed model. Further extensive simulation studies will be needed for assessing this.

Finally, we note that our proposed model assumes the meta-analysis data available is representative of the populations of interest (like all meta-analysis models). In particular, if asymmetry in the observed treatment effect estimates is due to bias, for example publication bias and selective reporting, then the summary result, the heterogeneity measures and the predictive inference may be inappropriate (as then the random effects distribution is inappropriately captured). Then, a concern may arise when it is difficult to distinguish possible causes of skewness on the observed treatment effect estimates. Several scenarios can be considered for the reason why the treatment effect estimates are skewed across studies; for example, (i) the treatment effect distribution suffers from the publication bias and/or the selective reporting, (ii) the treatment effect distribution is a mixture of two different distributions, (iii) the treatment effect distribution is truly skewed, (iv) the treatment effect distribution is skewed simply due to estimation errors. It should be performed first to explore the possible causes of skewness. When the scenario (i) or (ii) is true, our proposed model may not be appropriate, but is likely to be more robust than the normal random effects model. This is because extreme study results locating on one side are expressed as the tail of the treatment effect distribution in our proposed model. Therefore, we conclude our proposed model is applicable for all the scenarios and to is likley to produce more suitable meta-analysis results in comparison to the conventional normal random effects model. However, further research and extended simulations are needed to critically examine this in more detail, especially in situations where publication and selection biases are causing the asymmetry. Our proposed model aims to reduce non-normality in the random effects distribution by observing the non-normality in the overall distribution of the *y*_*i*_’s. Therefore, it is likely to perform best when the between-study heterogeneity is large relative to the within-study variability, such that skewness in the overall distribution can be detected and will be due to asymmetry in the true treatment effects. Further research may also consider applying the Box-Cox transformation to just the random effects distribution in model (1) (i.e. to just the *θ*_*i*_’s). Although Gurka et al. [15] suggests that the Box-Cox transformation in a mixed effects model should be viewed in terms of their success in normalising the total error, the *y*_*i*_ themselves do not need to be transformed, and thus left on their original scale familiar to meta-analysts.

## Conclusions

We proposed a random effects meta-analysis with Box-Cox transformation to deal with the skewness in meta-analysis data. The proposed meta-analysis model has the potential to provide more robust inferences for summary treatment effects when the random effects distribution is skewed. It could be used to examine the robustness of traditional meta-analysis results, heterogeneity measures, and predictive inferences to skewed random effects distributions. However, further research would be welcome to examine the method in further simulated and empirical examples.

## Additional files

Additional file 1 R code. Our Bayesian analyses in the simulation study and the application were implemented by using the R software. We provide R functions nremeta and bcremeta for the Bayesian estimations of the normal random effects model and the proposed model respectively. (PDF 45 kb)

Additional file 2 Results of the simulation study. We provide the following details related to designs and results of the simulation study: (i) density functions of the random effects distribution, (ii) full tables of the results, (iii) summary statistics of estimates for the transformation and the shift parameter. (PDF 2467 kb)

## Abbreviations

BC: Proposed model using Box-Cox transformation without sign inversion
BC-SI: Proposed model using Box-Cox transformation with sign inversion
EXP: Shifted exponential distribution
GLMMs: Generalised linear mixed models
IPD: Individual patient data
IQR: Interquartile range
LN: Shifted log-normal distribution
MCMC: Markov chain Monte Carlo
N: Normal distribution
NRE: Normal random effects model
nSN1: Skew-normal distribution with moderate negative skewness
nSN2: Skew-normal distribution with large negative skewness
pSN1: Skew-normal distribution with moderate positive skewness
pSN2: Skew-normal distribution with large positive skewness
RMSE: Root mean square error
