# Supplementary material for: A random effects meta-analysis model with Box-Cox transformation
Source: BMC Med Res Methodol. 2017 Jul 19;17:109. doi: 10.1186/s12874-017-0376-7 (PMC5517826; doi:10.1186/s12874-017-0376-7)
Supplement: Supplementary file 1 — R code. Our Bayesian analyses in the simulation study and the application were implemented by using the R software. We provide R functions nremeta and bcremeta for the Bayesian estimations of the normal random effects model and the proposed model respectively. (PDF 45 kb) [file 12874_2017_376_MOESM1_ESM.pdf]

Additional file 1 to "A random effects meta-analysis model with Box-Cox transformation"

1 R code

Our Bayesian analyses in the simulation study and the application were implemented by using the R software. In particular, we used rstan package which provides an interface to Stan. The Stan is a programming language for implementing Bayesian inference with the MCMC sampling. We below provide R functions `nremeta` and `bcremeta` for the Bayesian estimations of the normal random effects model and the proposed model respectively.

1.1 R function for normal random effects model: `nremeta`

Description: Function to implement Bayesian estimation of normal random effects model using MCMC sampling.

Usage: `nremeta(ns, yi, vi, iter.num, chain.num, burnin, thin)`

Arguments: `ns` = number of studies; `yi` = vector of observed treatment effect estimates; `vi` = vector of observed within-study variances for treatment effect estimates; `iter.num` = number of samples drawn within each chain of MCMC; `chain.num` = number of chains in MCMC; `burnin` = number of burn-in samples within each chain of MCMC; `thin` = thinning interval between consecutive samples within each chain of MCMC.

Returned list elements: `theta` = posterior mean, standard deviation (s.d.), median, lower and upper limit of 95 per cent credible interval for overall mean effect; `theta.samp` = samples of overall mean effect from its posterior distribution; `tau` = posterior mean, s.d., median, lower and upper limit of 95 per cent credible interval for square root of between-study variance; `I2` = posterior mean, s.d., median, lower and upper limit of 95 per cent credible interval for I^2; `pred` = mean, s.d. and median of samples from predictive distribution, and lower and upper limit of 95 per cent prediction interval; `pred.samp` = samples from predictive distribution.

Source code:

```
library(rstan)

make.df <- function(parsamp)
{
  no.parsamp <- na.omit(parsamp)
  dataf <- data.frame(
    mean=mean(no.parsamp), sd=sd(no.parsamp)
    ,median=quantile(no.parsamp,probs=0.500)[[1]]
    ,CIlower=quantile(no.parsamp,probs=0.025)[[1]]
    ,CIupper=quantile(no.parsamp,probs=0.975)[[1]]
  )
```

```
  return(dataf)
}

normal <- '
  data {
    int<lower=0> k;
    real yi[k];
    real<lower=0> vi[k];
  }
  parameters {
    real theta;
    real<lower=0> tau;
  }
  model {
    for(i in 1:k)
      yi[i] ~ normal(theta,sqrt(tau*tau+vi[i]));
    theta ~ normal(0,10000);
    tau ~ uniform(0,10);
  }'
nmeta <- stan(model_code=normal)

nremeta <- function(ns,yi,vi,iter.num,chain.num,burnin,thin)
{
  standata <- list(k=ns,yi=yi,vi=vi)
  res <- stan(fit=nmeta,data=standata,iter=iter.num,chains=chain.num,warmup=burnin,thin=thin)

  ntheta <- extract(res,par="theta",permuted=TRUE)$theta
  ntau <- extract(res,par="tau",permuted=TRUE)$tau

  wi <- 1/vi
  nsigma <- sum(wi*(ns-1))/(sum(wi)^2-sum(wi^2))
  nI2 <- ntau^2/(ntau^2+nsigma)*100
  npred <- rnorm(length(ntheta),mean=ntheta,sd=ntau)

  ntheta.df <- make.df(ntheta)
  ntau.df <- make.df(ntau)
  nI2.df <- make.df(nI2)
  npred.df <- make.df(npred)
  ntheta2.df <- data.frame(samples=ntheta)
  npred2.df <- data.frame(samples=npred)

  return(list(
    theta=ntheta.df,theta.samp=ntheta2.df,tau=ntau.df
    ,I2=nI2.df,pred=npred.df,pred.samp=npred2.df))
}
```

1.2 R function for random effects model with Box-Cox transformation: `bcremeta`

Description: Function to implement Bayesian estimation of random effects model with Box-Cox transformation using MCMC sampling.

Usage: `bcremeta(ns, yi, vi, grid.alpmin, grid.lambda, iter.num, chain.num, burnin, thin)`

Arguments: `ns` = number of studies; `yi` = vector of observed treatment effect estimates; `vi` = vector of observed within-study variances for treatment effect estimates; `grid.alphmin` = vector of candedate values of grid search procedure for estimating shift parameter, which is specified as minimum values of $\{(y_i + \alpha) : i = 1, \ldots, k\}$; `grid.lambda` = vector of candedate values of grid search procedure for estimating transformation parameter; `iter.num` = number of samples drawn within each chain of MCMC; `chain.num` = number of chains in MCMC; `burnin` = number of burn-in samples within each chain of MCMC; `thin` = thinning interval between consecutive samples within each chain of MCMC.

Returned list elements: `median` = posterior mean, standard deviation (s.d.), median, lower and upper limit of 95 per cent credible interval for overall median effect; `median.samp` = samples of overall median effect from its posterior distribution; `IQR` = posterior mean, s.d., median, lower and upper limit of 95 per cent credible interval for IQR of random effects distribution ($\xi_{75} - \xi_{25}$); `nIQR` = posterior mean, s.d., median, lower and upper limit of 95 per cent credible interval for normalised IQR of random effects distribution ($(\xi_{75} - \xi_{25})/(z_{75} - z_{25})$); `IQRrs` = posterior mean, s.d., median, lower and upper limit of 95 per cent credible interval for ratio of IQR squares ($(\xi_{75} - \xi_{25})^2/(\nu_{75} - \nu_{25})^2$); `pred` = mean, s.d. and median of samples from predictive distribution, and lower and upper limit of 95 per cent prediction interval; `pred.samp` = samples from predictive distribution; `tau` = posterior mean, s.d., median, lower and upper limit of 95 per cent credible interval for square root of between-study variance of random effects after transformation; `lambda` = estimate of transformation parameter; `alpha` = estimate of shift parameter; `alpmin` = estimate of $\min\{(y_i + \alpha) : i = 1, \ldots, k\}$.

Source code:

```
library(rstan)

make.df <- function(parsamp)
{
  no.parsamp <- na.omit(parsamp)
  dataf <- data.frame(
    mean=mean(no.parsamp), sd=sd(no.parsamp)
    ,median=quantile(no.parsamp,probs=0.500)[[1]]
    ,CIlower=quantile(no.parsamp,probs=0.025)[[1]]
    ,CIupper=quantile(no.parsamp,probs=0.975)[[1]]
  )
  return(dataf)
}

bc.like <- function(k,y,s2,lam,alp) { return(
  function(para) {
    mu <- para[1]
    tau <- para[2]
    ygm <- prod(y+alp)^(1/k)
    yl <- ((y+alp)^lam-1)/(lam*ygm^(lam-1))
    phi2 <- s2/ygm^(2*lam-2)*(lam*ygm^(lam-1)*mu+1)^(2-2/lam)
    return(sum(-0.5*log(tau^2+phi2)-0.5/(tau^2+phi2)*(yl-mu)^2))
  }
```

```
)}

boxcox <- '
  data {
    int<lower=0> k;
    real yi[k];
    real ygm;
    real<lower=0> vi[k];
    real lambda;
    real alpha;
  }
  parameters {
    real mu;
    real<lower=0> tau;
  }
  model {
    real ylami;
    real phi2;
    for (i in 1:k) {
      ylami <- (pow(yi[i]+alpha,lambda)-1)/(lambda*pow(ygm,lambda-1));
      phi2 <- vi[i]/pow(ygm,2*lambda-2)*pow(lambda*pow(ygm,lambda-1)*mu+1,2-2/lambda);
      increment_log_prob(-0.5*log(tau*tau+phi2)-0.5/(tau*tau+phi2)*pow(ylami-mu,2));
    }
    mu ~ normal(0,10000);
    tau ~ uniform(0,10);
  }'
bcmeta <- stan(model_code=boxcox)

bcremeta <- function(ns,yi,vi,grid.alpmin,grid.lambda,iter.num,chain.num,burnin,thin)
{
  glambda <- grid.lambda[grid.lambda!=0]

  if(min(yi)>0) {

    mutau <- array(0,dim=c(4,length(glambda)))
    alpha <- 0
    ygmean <- prod(yi)^(1/ns)
    transf <- apply(as.matrix(glambda),1,function(a) {
      return((yi^a-1)/(a*ygmean^(a-1)))
    })
    transf.niqr <- apply(transf,2,function(a) {
      nq3 <- qnorm(0.75,0,1)
      nq1 <- qnorm(0.25,0,1)
      return(IQR(a)/(nq3-nq1))
    })
    bcox <- cbind(apply(transf,2,median),transf.niqr)

    for(l in 1:length(glambda)) {
      lambda <- glambda[l]
      if(lambda>0) {
        bclim <- -1/(lambda*ygmean^(lambda-1))
```

```
      low <- c(bclim+0.01,0)
      upp <- c(Inf,Inf)
    } else {
      bclim <- -1/(lambda*ygmean^(lambda-1))
      low <- c(-Inf,0)
      upp <- c(bclim-0.01,Inf)
    }

    like.init <- as.vector(bcox[l,]+0.001)
    for(ll in 1:10) {
      tmp <- try(optim(
              par=like.init, fn=bc.like(ns,yi,vi,lambda,alpha),control=list(fnscale=-1)
              ,method="L-BFGS-B",lower=low,upper=upp),TRUE)

      if(class(tmp)=="try-error") {
        if(ll==10) {
          mutau[,l] <- c(0,0,lambda,-99999)
          break
        } else {
          like.init <- like.init+0.001
        }
      } else {
        mutau[,l] <- c(tmp$par,lambda,tmp$value)
        break
      }
    }
  }
  mle <- c(mutau[1:3,order(-mutau[4,])[1]],alpha)

} else {

  mutau <- array(0,dim=c(4,length(glambda)))
  pmle <- array(0,dim=c(5,length(grid.alpmin)))

  for(m in 1:length(grid.alpmin)) {
    alpha <- grid.alpmin[m]-min(yi)
    ygmean <- prod(yi+alpha)^(1/ns)
    transf <- apply(as.matrix(glambda),1,function(a) {
      return(((yi+alpha)^a-1)/(a*ygmean^(a-1)))
    })
    transf.niqr <- apply(transf,2,function(a) {
      nq3 <- qnorm(0.75,0,1)
      nq1 <- qnorm(0.25,0,1)
      return(IQR(a)/(nq3-nq1))
    })
    bcox <- cbind(apply(transf,2,median),transf.niqr)

    for(l in 1:length(glambda)) {
      lambda <- glambda[l]
      if(lambda>0) {
        bclim <- -1/(lambda*ygmean^(lambda-1))
```

```
        low <- c(bclim+0.01,0)
        upp <- c(Inf,Inf)
      } else {
        bclim <- -1/(lambda*ygmean^(lambda-1))
        low <- c(-Inf,0)
        upp <- c(bclim-0.01,Inf)
      }

      like.init <- as.vector(bcox[1,]+0.001)
      for(ll in 1:10) {
        tmp <- try(optim(
                par=like.init,fn=bc.like(ns,yi,vi,lambda,alpha),control=list(fnscale=-1)
                ,method="L-BFGS-B",lower=low,upper=upp),TRUE)

        if(class(tmp)=="try-error") {
          if(ll==10) {
            mutau[,l] <- c(0,0,lambda,-99999)
            break
          } else {
            like.init <- like.init+0.001
          }
        } else {
          mutau[,l] <- c(tmp$par,lambda,tmp$value)
          break
        }
      }
    }
    pmle[,m] <- c(mutau[,order(-mutau[4,])[1]],alpha)
  }

  mle <- pmle[c(1,2,3,5),order(-pmle[4,])[1]]
}

bclambda <- mle[3]
bcalpha <- mle[4]
bcalphmin <- bcalpha+min(yi)
ygmean <- prod(yi+bcalpha)^(1/ns)

standata <- list(k=ns,yi=yi,ygm=ygmean,vi=vi,lambda=bclambda,alpha=bcalpha)
init.list <- rep(list(list(mu=mle[1],tau=mle[2]+0.01)),chain.num)
res <- stan(fit=bcmeta,data=standata,iter=iter.num,chains=chain.num
        ,init=init.list,warmup=burnin,thin=thin)

bcmu <- extract(res,par="mu",permuted=TRUE)$mu
bctau <- extract(res,par="tau",permuted=TRUE)$tau

bcmed <- (bclambda*ygmean^(bclambda-1)*bcmu+1)^(1/bclambda)-bcalpha
bcxi25 <- (bclambda*ygmean^(bclambda-1)*(bcmu+bctau*qnorm(0.25,0,1))+1)^(1/bclambda)-bcalpha
bcxi75 <- (bclambda*ygmean^(bclambda-1)*(bcmu+bctau*qnorm(0.75,0,1))+1)^(1/bclambda)-bcalpha

bcIQR <- bcxi75-bcxi25
```

```
  bcNIQR <- (bcxi75-bcxi25)/(qnorm(0.75,0,1)-qnorm(0.25,0,1))

  bcsigma <- apply(as.matrix(bcmu),1,function(a) {
    phi2 <- vi/ygmean^(2*bclambda-2)*(bclambda*ygmean^(bclambda-1)*a+1)^(2-2/bclambda)
    wi <- 1/phi2
    return(sum(wi*(ns-1))/(sum(wi)^2-sum(wi^2)))
  })

  bctsd <- sqrt(bctau^2+bcsigma)
  bcnu25 <- (bclambda*ygmean^(bclambda-1)*(bcmu+bctsd*qnorm(0.25,0,1))+1)^(1/bclambda)-bcalpha
  bcnu75 <- (bclambda*ygmean^(bclambda-1)*(bcmu+bctsd*qnorm(0.75,0,1))+1)^(1/bclambda)-bcalpha
  bcIQRrs <- (bcxi75-bcxi25)^2/(bcnu75-bcnu25)^2*100

  bsamp1 <- rnorm(length(bcmu),mean=bcmu,sd=bctau)
  if(bclambda<0)
    bsamp2 <- subset(bsamp1,bsamp1<(-1/(bclambda*ygmean^(bclambda-1))))
  } else {
    bsamp2 <- subset(bsamp1,bsamp1>(-1/(bclambda*ygmean^(bclambda-1))))
  }
  bcpred <- (bclambda*ygmean^(bclambda-1)*bsamp2+1)^(1/bclambda)-bcalpha

  bcmed.df <- make.df(bcmed)
  bcIQR.df <- make.df(bcIQR)
  bcNIQR.df <- make.df(bcNIQR)
  bcIQRrs.df <- make.df(bcIQRrs)
  bcpred.df <- make.df(bcpred)
  bctau.df <- make.df(bctau)
  bcmed2.df <- data.frame(samples=bcmed)
  bcpred2.df <- data.frame(samples=bcpred)

  return(list(
    median=bcmed.df,median.samp=bcmed2.df,IQR=bcIQR.df,nIQR=bcNIQR.df
    ,IQRrs=bcIQRrs.df,pred=bcpred.df,pred.samp=bcpred2.df,tau=bctau.df
    ,lambda=bclambda,alpha=bcalpha,alpmin=bcalphmin))
}
```
