# Supplementary material for: A random effects meta-analysis model with Box-Cox transformation
Source: BMC Med Res Methodol. 2017 Jul 19;17:109. doi: 10.1186/s12874-017-0376-7 (PMC5517826; doi:10.1186/s12874-017-0376-7)
Supplement: Supplementary file 2 — Results of the simulation study. We provide the following details related to designs and results of the simulation study: (i) density functions of the random effects distribution, (ii) full tables of the results, (iii) summary statistics of estimates for the transformation and the shift parameter. (PDF 2467 kb) [file 12874_2017_376_MOESM2_ESM.pdf]

Additional file 2 to "A random effects meta-analysis model with Box-Cox transformation"

1 Random effects distributions in the simulation study

In the simulation study, we considered a variety of scenarios for the random effects distributions (normal distribution, skew-normal distribution, shifted exponential distribution and shifted log-normal distribution) which a true treatment effect θ_i for the ith study was drawn from. Here, the random effects distributions are graphically illustrated for each scenario. Figure S1, Figure S2, Figure S3, Figure S4, Figure S5, Figure S6 and Figure S7 show density functions of the random effects distribution for the scenarios 1-3, 4-6, 7-9, 10-12, 13-15, 16-18 and 19-21, respectively.

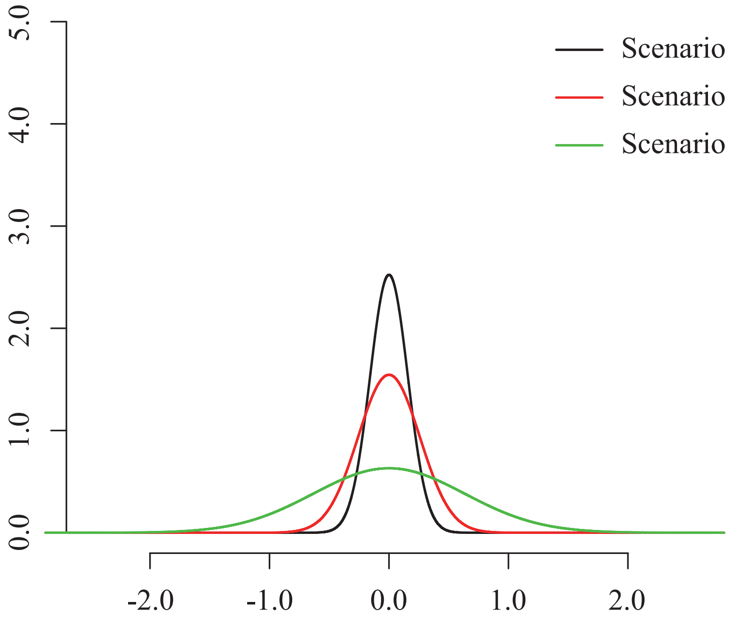

Figure S1. Density functions of the random effects distributions (Scenario 1-3: Normal distribution).

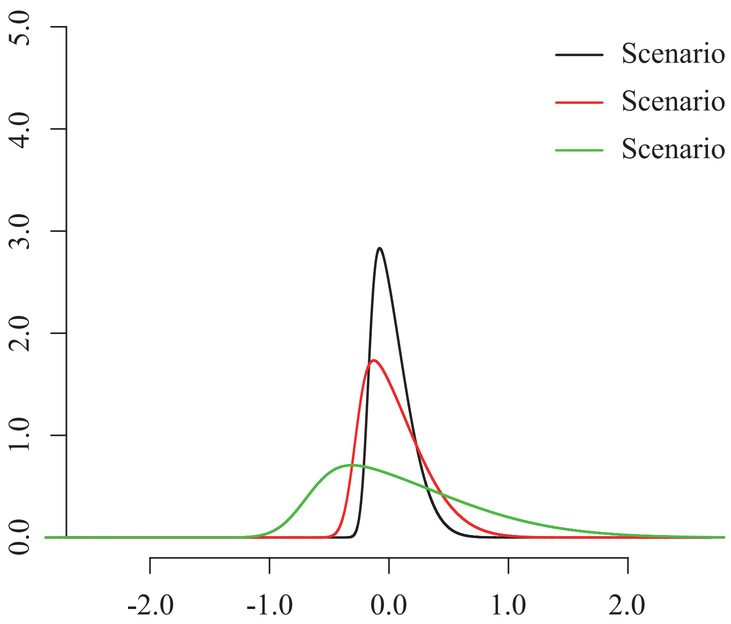

Figure S2. Density functions of the random effects distributions (Scenario 4-6: Skew-normal distribution with moderate positive skewness).

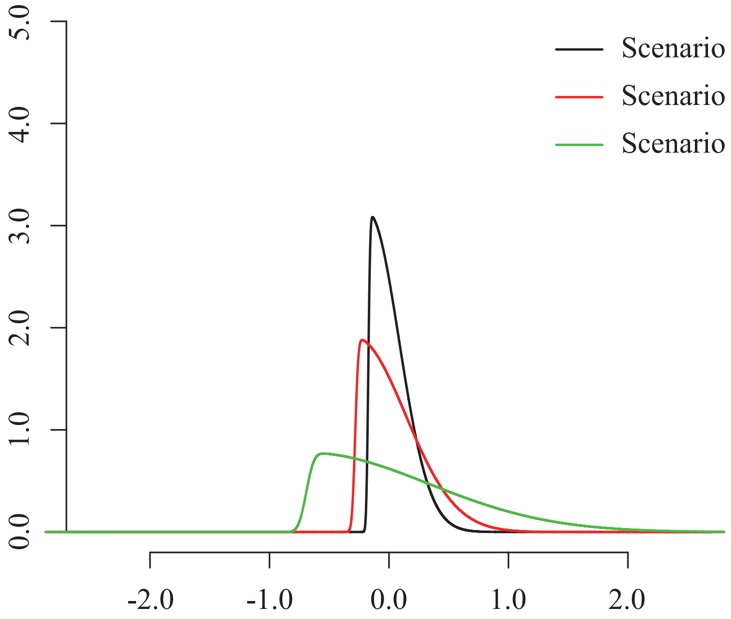

Figure S3. Density functions of the random effects distributions (Scenario 7-9: Skew-normal distribution with large positive skewness).

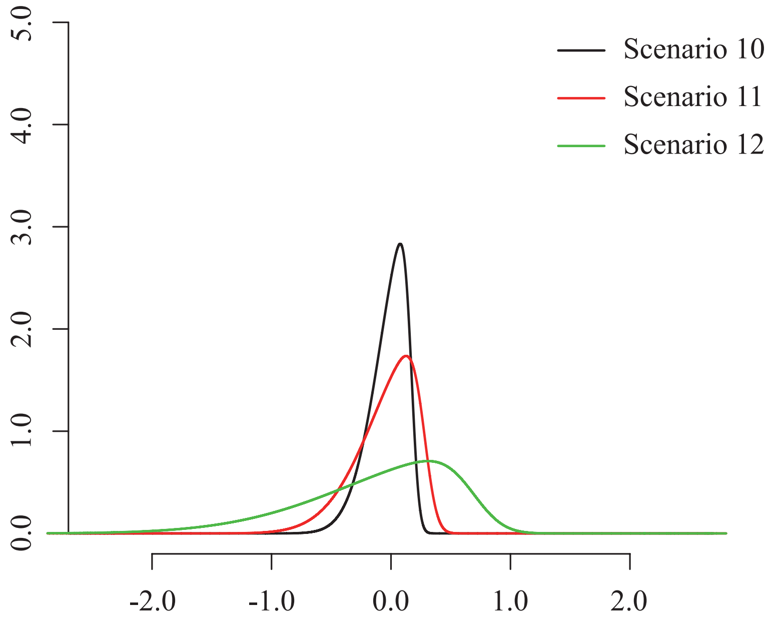

Figure S4. Density functions of the random effects distributions (Scenario 10-12: Skew-normal distribution with moderate negative skewness).

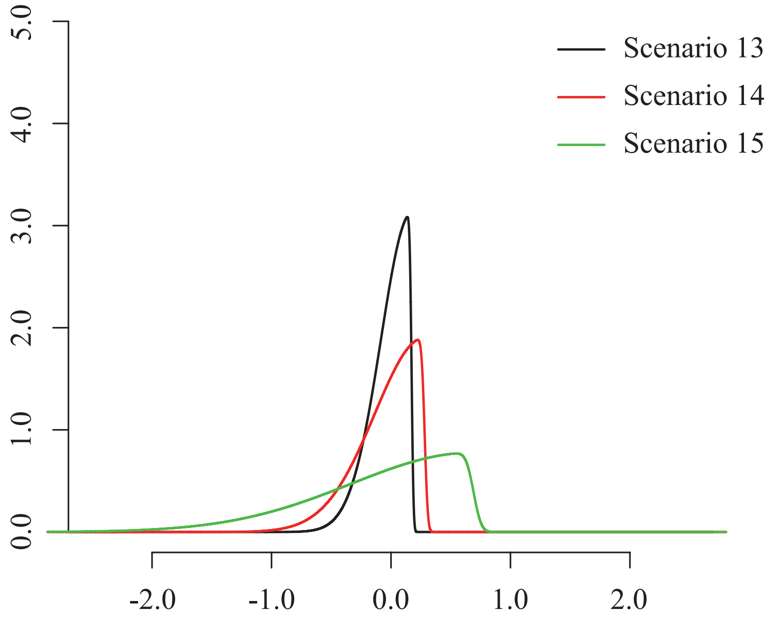

Figure S5. Density functions of the random effects distributions (Scenario 13-15: Skew-normal distribution with large negative skewness).

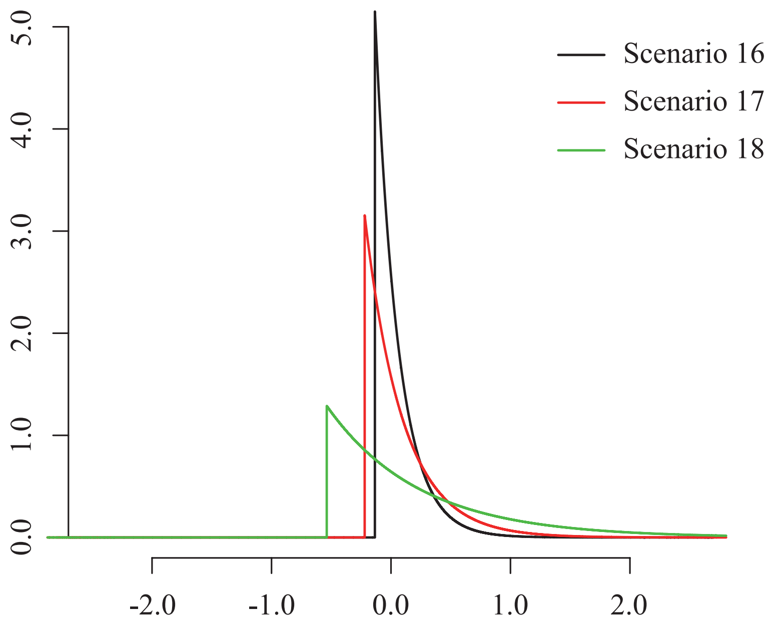

Figure S6. Density functions of the random effects distributions (Scenario 16-18: Shifted exponential distribution).

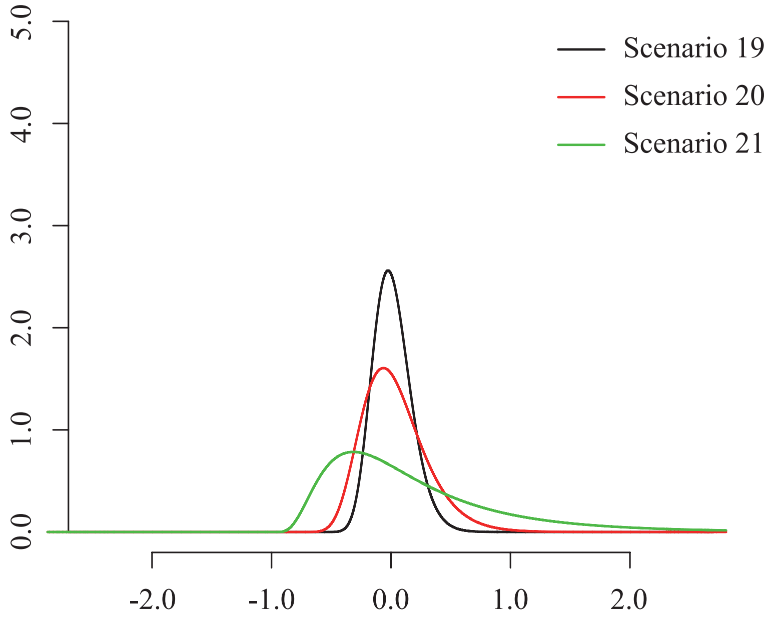

Figure S7. Density functions of the random effects distributions (Scenario 19-21: Shifted log-normal distribution).

2 Results of the simulation study

Table S1, Table S2, Table S3 and Table S4 show results from the two models, for each scenario of the number of studies k = 5, 10, 20 and 40, respectively.

Table S1. Bias, root mean square error, coverage probability from the two models for scenario of the number of studies $k = 5$.

		Bias			RMSE			Coverage prob. (%)		
Scenario	True	NRE	BC	BC-SI	NRE	BC	BC-SI	NRE	BC	BC-SI
Scenario 1-3: Normal distribution (N)										
Overall mean or overall median effect										
1	0.000	0.000	−0.030	0.000	0.158	0.176	0.177	99.5	93.5	90.9
2	0.000	0.000	−0.047	0.001	0.184	0.210	0.213	99.1	93.8	91.5
3	0.000	0.000	−0.130	0.004	0.316	0.379	0.397	97.4	94.6	93.8
I^2 or ratio of IQR squares (%)										
1	20.0	23.54	23.51	25.22	29.88	28.94	29.58	97.5	97.1	98.6
2	40.0	10.71	9.05	10.52	23.05	21.03	20.21	96.5	96.2	98.1
3	80.0	−2.47	−7.09	−7.21	18.57	21.44	19.90	94.1	91.7	97.8
Scenario 4-6: Skew-normal distribution with moderate positive skewness (pSN1)										
Overall mean or overall median effect										
4	0.000	0.025	−0.008	0.024	0.159	0.169	0.176	99.5	92.0	90.3
5	0.000	0.043	−0.011	0.033	0.188	0.196	0.212	99.1	92.5	91.2
6	0.000	0.110	−0.054	0.051	0.333	0.325	0.384	97.6	93.9	93.6
I^2 or ratio of IQR squares (%)										
4	20.0	23.59	23.80	25.34	29.90	29.18	29.57	97.4	96.9	98.7
5	40.0	10.91	9.45	10.68	23.26	21.28	20.27	96.9	96.3	98.2
6	80.0	-3.89	-8.99	-8.86	19.94	22.22	21.02	93.6	94.3	98.1
Scenario 7-9: Skew-normal distribution with large positive skewness (pSN2)										
Overall mean or overall median effect										
7	0.000	0.032	−0.001	0.028	0.160	0.172	0.178	99.6	91.1	89.8
8	0.000	0.054	0.000	0.045	0.192	0.196	0.216	99.1	91.4	90.8
9	0.000	0.125	−0.045	0.055	0.330	0.313	0.380	98.0	94.2	94.0
I^2 or ratio of IQR squares (%)										
7	20.0	23.29	23.12	24.98	29.58	28.51	29.21	97.7	97.2	98.9
8	40.0	10.37	9.10	10.21	22.95	20.76	19.89	96.5	96.7	98.3
9	80.0	−3.81	−9.10	−8.94	19.75	22.25	21.22	93.8	94.7	97.6

RMSE: root mean square error, prob.: probability
NRE: normal random effects model, BC: proposed model using Box-Cox transformation
BC-SI: proposed model using Box-Cox transformation with the sign inversion for negatively skewed data

Table S1 (cont'd). Bias, root mean square error, coverage probability from the two models for scenario of the number of studies $k = 5$.

		Bias			RMSE			Coverage prob. (%)		
Scenario	True	NRE	BC	BC-SI	NRE	BC	BC-SI	NRE	BC	BC-SI
Scenario 10-12: Skew-normal distribution with moderate negative skewness (nSN1)										
Overall mean or overall median effect										
10	0.000	−0.027	−0.057	−0.024	0.161	0.186	0.180	99.6	94.7	90.9
11	0.000	−0.047	−0.089	−0.038	0.188	0.225	0.213	99.0	95.2	91.9
12	0.000	−0.110	−0.201	−0.048	0.341	0.432	0.392	97.4	95.0	93.5
I^2 or ratio of IQR squares (%)										
10	20.0	23.62	23.69	25.25	30.02	29.13	29.68	97.1	96.9	98.7
11	40.0	10.72	8.98	10.33	23.26	21.08	20.23	96.4	96.1	98.0
12	80.0	−3.57	−8.58	−8.41	19.80	22.94	20.94	93.5	89.4	97.9
Scenario 13-15: Skew-normal distribution with large negative skewness (nSN2)										
Overall mean or overall median effect										
13	0.000	−0.033	−0.061	−0.030	0.160	0.185	0.177	99.4	94.7	90.2
14	0.000	−0.047	−0.088	−0.039	0.187	0.224	0.212	99.2	94.8	90.8
15	0.000	−0.129	−0.213	−0.056	0.341	0.438	0.383	97.5	95.4	93.7
I^2 or ratio of IQR squares (%)										
13	20.0	23.12	23.14	24.87	29.39	28.50	29.18	97.7	97.1	98.9
14	40.0	10.45	8.75	10.31	22.88	20.73	19.84	96.7	96.1	98.3
15	80.0	−4.07	−9.40	−9.23	20.04	23.11	21.28	93.7	89.6	97.9
Scenario 16-18: Shifted exponential distribution (EXP)										
Overall mean or overall median effect										
16	0.000	0.062	0.021	0.053	0.177	0.176	0.189	99.4	90.3	90.0
17	0.000	0.096	0.029	0.070	0.223	0.201	0.228	98.9	89.6	89.9
18	0.000	0.237	0.014	0.094	0.446	0.319	0.400	98.2	92.9	92.7
I^2 or ratio of IQR squares (%)										
16	20.0	25.66	25.23	26.64	32.17	30.69	31.11	95.5	96.7	98.3
17	40.0	13.23	10.98	11.92	25.90	22.89	22.13	91.6	95.4	96.9
18	80.0	−2.91	−8.53	−8.53	21.26	22.65	21.88	83.2	94.4	96.7
Scenario 19-21: Shifted log-normal distribution (LN)										
Overall mean or overall median effect										
19	0.000	0.013	−0.021	0.011	0.157	0.173	0.177	99.7	93.5	91.6
20	0.000	0.035	−0.021	0.026	0.192	0.203	0.217	99.2	92.6	91.3
21	0.000	0.211	−0.012	0.073	0.448	0.333	0.401	98.2	93.3	93.0
I^2 or ratio of IQR squares (%)										
19	20.0	24.10	24.14	25.68	30.40	29.56	29.98	97.3	96.9	98.8
20	40.0	11.53	9.86	10.99	23.68	21.62	20.66	96.0	96.5	98.0
21	80.0	−1.72	−7.48	−7.38	20.13	22.07	21.10	83.3	93.6	96.7

RMSE: root mean square error, prob.: probability
NRE: normal random effects model, BC: proposed model using Box-Cox transformation
BC-SI: proposed model using Box-Cox transformation with the sign inversion for negatively skewed data

Table S2. Bias, root mean square error, coverage probability from the two models for scenario of the number of studies $k = 10$.

Scenario	True	Bias			RMSE			Coverage prob. (%)		
		NRE	BC	BC-SI	NRE	BC	BC-SI	NRE	BC	BC-SI
Scenario 1-3: Normal distribution (N)										
Overall mean or overall median effect										
1	0.000	0.001	−0.026	0.000	0.112	0.125	0.129	98.0	96.2	95.8
2	0.000	0.000	−0.038	0.000	0.131	0.150	0.156	97.1	95.9	95.7
3	0.000	−0.001	−0.079	0.000	0.228	0.266	0.281	95.5	94.9	95.4
I^2 or ratio of IQR squares (%)										
1	20.0	10.77	10.99	12.38	19.95	19.78	20.27	97.9	97.6	98.1
2	40.0	1.54	0.95	2.27	19.75	19.61	19.11	96.9	96.8	97.4
3	80.0	−1.90	−3.52	−2.57	13.84	16.16	15.01	93.1	92.1	94.7
Scenario 4-6: Skew-normal distribution with moderate positive skewness (pSN1)										
Overall mean or overall median effect										
4	0.000	0.025	−0.005	0.020	0.116	0.122	0.132	97.5	95.6	95.5
5	0.000	0.043	−0.004	0.027	0.139	0.143	0.158	96.2	95.1	94.8
6	0.000	0.110	−0.020	0.029	0.254	0.238	0.272	95.2	95.2	95.1
I^2 or ratio of IQR squares (%)										
4	20.0	10.31	10.74	11.92	19.59	19.50	19.83	98.0	97.6	98.3
5	40.0	1.28	0.98	1.93	19.88	19.61	19.10	96.7	96.9	97.5
6	80.0	−2.89	−4.57	−4.21	15.14	16.98	16.29	92.0	94.1	95.9
Scenario 7-9: Skew-normal distribution with large positive skewness (pSN2)										
Overall mean or overall median effect										
7	0.000	0.032	0.002	0.027	0.117	0.122	0.133	97.4	95.6	95.2
8	0.000	0.050	0.003	0.034	0.139	0.140	0.157	96.4	95.1	94.9
9	0.000	0.127	−0.010	0.035	0.257	0.230	0.265	95.4	95.5	95.4
I^2 or ratio of IQR squares (%)										
7	20.0	10.54	10.99	12.11	19.94	19.90	20.19	97.8	97.6	98.1
8	40.0	0.91	0.58	1.47	19.84	19.58	19.01	96.6	97.0	97.5
9	80.0	−3.78	−5.81	−5.55	16.07	18.02	17.42	91.5	94.7	96.3

RMSE: root mean square error, prob.: probability
NRE: normal random effects model, BC: proposed model using Box-Cox transformation
BC-SI: proposed model using Box-Cox transformation with the sign inversion for negatively skewed data

Table S2 (cont'd). Bias, root mean square error, coverage probability from the two models for scenario of the number of studies $k = 10$.

Scenario	True	Bias			RMSE			Coverage prob. (%)		
		NRE	BC	BC-SI	NRE	BC	BC-SI	NRE	BC	BC-SI
Scenario 10-12: Skew-normal distribution with moderate negative skewness (nSN1)										
Overall mean or overall median effect										
10	0.000	−0.027	−0.052	-0.023	0.116	0.134	0.131	97.6	96.3	95.9
11	0.000	−0.044	−0.071	-0.030	0.138	0.162	0.157	96.5	95.0	95.2
12	0.000	−0.111	−0.126	-0.026	0.252	0.293	0.270	95.5	94.0	95.2
I^2 or ratio of IQR squares (%)										
10	20.0	10.70	10.90	12.31	19.96	19.65	20.21	97.6	97.5	98.1
11	40.0	1.40	0.08	1.99	20.02	19.69	19.28	96.8	96.7	97.5
12	80.0	−2.70	−6.08	−4.20	14.99	18.10	16.31	91.7	91.5	95.8
Scenario 13-15: Skew-normal distribution with large negative skewness (nSN2)										
Overall mean or overall median effect										
13	0.000	−0.031	−0.055	−0.027	0.116	0.134	0.130	97.4	96.0	95.2
14	0.000	−0.052	−0.075	−0.036	0.138	0.161	0.154	96.3	95.0	95.1
15	0.000	−0.123	−0.130	−0.029	0.256	0.293	0.265	95.0	93.5	94.9
I^2 or ratio of IQR squares (%)										
13	20.0	10.24	10.49	11.88	19.55	19.32	19.79	98.1	97.8	98.4
14	40.0	0.66	−0.78	1.23	19.84	19.45	18.97	96.5	96.8	97.4
15	80.0	−3.80	−7.54	−5.49	15.92	19.10	17.06	91.6	91.8	96.4
Scenario 16-18: Shifted exponential distribution (EXP)										
Overall mean or overall median effect										
16	0.000	0.059	0.020	0.044	0.133	0.126	0.140	96.7	94.9	94.5
17	0.000	0.096	0.030	0.057	0.172	0.145	0.166	95.3	94.8	94.4
18	0.000	0.240	0.038	0.064	0.359	0.238	0.265	94.9	96.1	95.7
I^2 or ratio of IQR squares (%)										
16	20.0	13.89	13.81	14.80	23.35	22.62	22.88	94.6	95.5	96.2
17	40.0	6.61	5.05	5.75	23.10	21.84	21.51	89.9	93.6	94.2
18	80.0	−0.39	−4.21	−4.09	16.70	18.14	17.81	75.5	91.7	92.8
Scenario 19-21: Shifted log-normal distribution (LN)										
Overall mean or overall median effect										
19	0.000	0.012	−0.017	0.010	0.115	0.124	0.131	97.8	96.1	95.6
20	0.000	0.035	−0.012	0.022	0.137	0.144	0.157	96.7	95.6	95.4
21	0.000	0.207	0.012	0.043	0.345	0.240	0.266	95.9	96.4	96.3
I^2 or ratio of IQR squares (%)										
19	20.0	10.89	11.18	12.41	20.11	19.87	20.31	97.9	97.4	98.1
20	40.0	2.59	1.89	2.99	20.23	19.92	19.47	95.8	96.2	96.9
21	80.0	0.43	−2.76	−2.51	15.68	17.04	16.58	75.5	90.6	91.7

RMSE: root mean square error, prob.: probability
NRE: normal random effects model, BC: proposed model using Box-Cox transformation
BC-SI: proposed model using Box-Cox transformation with the sign inversion for negatively skewed data

Table S3. Bias, root mean square error, coverage probability from the two models for scenario of the number of studies $k = 20$.

Scenario	True	Bias			RMSE			Coverage prob. (%)		
		NRE	BC	BC-SI	NRE	BC	BC-SI	NRE	BC	BC-SI
Scenario 1-3: Normal distribution (N)										
Overall mean or overall median effect										
1	0.000	−0.001	−0.017	0.000	0.080	0.089	0.091	96.6	94.6	94.7
2	0.000	0.001	−0.020	0.000	0.093	0.105	0.109	95.9	93.8	93.6
3	0.000	0.001	−0.038	0.001	0.160	0.182	0.189	95.4	93.6	94.0
I^2 or ratio of IQR squares (%)										
1	20.0	3.99	4.33	5.94	15.11	15.45	16.17	98.1	97.7	97.5
2	40.0	−1.84	−1.96	0.15	17.69	18.22	17.86	96.9	96.6	96.2
3	80.0	−0.83	−0.95	0.22	8.40	9.27	8.29	93.3	92.2	93.0
Scenario 4-6: Skew-normal distribution with moderate positive skewness (pSN1)										
Overall mean or overall median effect										
4	0.000	0.026	0.007	0.022	0.085	0.087	0.094	95.5	94.8	93.7
5	0.000	0.045	0.013	0.029	0.104	0.102	0.113	93.7	93.8	92.6
6	0.000	0.109	0.010	0.027	0.193	0.167	0.181	92.9	94.8	93.9
I^2 or ratio of IQR squares (%)										
4	20.0	3.75	4.31	5.67	15.09	15.59	16.12	97.8	97.3	97.2
5	40.0	−1.80	−1.30	−0.04	18.02	18.42	18.07	95.9	95.9	95.9
6	80.0	−1.12	−1.16	−0.89	9.25	9.77	9.32	91.3	92.9	93.6
Scenario 7-9: Skew-normal distribution with large positive skewness (pSN2)										
Overall mean or overall median effect										
7	0.000	0.030	0.011	0.026	0.085	0.086	0.094	95.4	95.0	94.0
8	0.000	0.051	0.018	0.033	0.106	0.101	0.111	92.8	93.6	92.3
9	0.000	0.125	0.018	0.033	0.201	0.165	0.179	91.6	94.7	93.3
I^2 or ratio of IQR squares (%)										
7	20.0	3.24	3.87	5.19	14.80	15.25	15.77	98.2	97.8	97.8
8	40.0	−3.15	−2.74	−1.53	18.24	18.54	18.13	96.6	96.5	96.6
9	80.0	−2.05	−2.40	−2.23	9.97	10.58	10.31	91.6	94.1	94.5

RMSE: root mean square error, prob.: probability
NRE: normal random effects model, BC: proposed model using Box-Cox transformation
BC-SI: proposed model using Box-Cox transformation with the sign inversion for negatively skewed data

Table S3 (cont'd). Bias, root mean square error, coverage probability from the two models for scenario of the number of studies $k = 20$.

		Bias			RMSE			Coverage prob. (%)		
Scenario	True	NRE	BC	BC-SI	NRE	BC	BC-SI	NRE	BC	BC-SI
Scenario 10-12: Skew-normal distribution with moderate negative skewness (nSN1)										
Overall mean or overall median effect										
10	0.000	−0.028	−0.041	−0.024	0.085	0.096	0.093	95.6	93.1	93.7
11	0.000	−0.046	−0.054	−0.031	0.103	0.115	0.111	94.1	91.8	93.0
12	0.000	−0.109	−0.080	−0.029	0.195	0.199	0.183	92.5	90.8	93.7
I^2 or ratio of IQR squares (%)										
10	20.0	4.03	4.09	5.93	15.22	15.46	16.25	97.8	97.4	97.1
11	40.0	−2.01	−3.25	−0.22	17.94	18.28	17.96	96.2	96.8	96.2
12	80.0	−1.23	−4.04	−0.94	9.32	11.33	9.24	91.0	92.4	93.5
Scenario 13-15: Skew-normal distribution with large negative skewness (nSN2)										
Overall mean or overall median effect										
13	0.000	−0.032	−0.044	−0.027	0.086	0.097	0.095	95.2	92.7	93.2
14	0.000	−0.050	−0.057	−0.035	0.105	0.117	0.111	93.5	90.8	92.5
15	0.000	−0.127	−0.089	−0.036	0.202	0.199	0.179	91.5	89.8	93.3
I^2 or ratio of IQR squares (%)										
13	20.0	3.36	3.34	5.28	14.74	14.86	15.74	98.1	98.0	97.8
14	40.0	−3.31	−4.77	−1.65	18.12	18.55	18.03	96.7	97.2	96.8
15	80.0	−2.10	−5.84	−2.24	10.02	12.90	10.20	91.5	91.3	94.4
Scenario 16-18: Shifted exponential distribution (EXP)										
Overall mean or overall median effect										
16	0.000	0.059	0.032	0.045	0.103	0.094	0.103	91.9	93.3	91.5
17	0.000	0.095	0.044	0.055	0.139	0.111	0.120	89.1	92.9	91.8
18	0.000	0.238	0.070	0.076	0.304	0.184	0.190	85.0	94.7	94.1
I^2 or ratio of IQR squares (%)										
16	20.0	7.87	8.28	9.37	18.51	18.73	19.20	94.0	94.3	94.1
17	40.0	5.11	4.21	4.94	20.61	20.14	19.95	86.1	89.8	89.8
18	80.0	2.31	−0.66	−0.60	10.99	11.39	11.24	69.4	87.8	88.1
Scenario 19-21: Shifted log-normal distribution (LN)										
Overall mean or overall median effect										
19	0.000	0.013	−0.006	0.010	0.082	0.088	0.093	96.4	94.9	94.3
20	0.000	0.034	0.001	0.018	0.101	0.102	0.110	94.4	94.3	93.6
21	0.000	0.208	0.041	0.048	0.286	0.177	0.183	90.0	95.5	94.9
I^2 or ratio of IQR squares (%)										
19	20.0	4.19	4.69	6.13	15.36	15.77	16.44	97.9	97.6	97.4
20	40.0	−0.23	0.04	1.37	18.04	18.47	18.16	95.1	94.9	94.9
21	80.0	3.35	1.00	1.08	10.58	10.36	10.18	66.4	85.0	85.3

RMSE: root mean square error, prob.: probability
NRE: normal random effects model, BC: proposed model using Box-Cox transformation
BC-SI: proposed model using Box-Cox transformation with the sign inversion for negatively skewed data

Table S4. Bias, root mean square error, coverage probability from the two models for scenario of the number of studies $k = 40$.

		Bias			RMSE			Coverage prob. (%)		
Scenario	True	NRE	BC	BC-SI	NRE	BC	BC-SI	NRE	BC	BC-SI
Scenario 1-3: Normal distribution (N)										
Overall mean or overall median effect										
1	0.000	0.000	−0.009	0.000	0.056	0.062	0.064	95.8	94.1	93.7
2	0.000	0.000	−0.010	0.000	0.066	0.073	0.075	94.8	92.8	93.1
3	0.000	0.000	−0.019	0.001	0.113	0.126	0.130	95.0	93.3	93.1
I^2 or ratio of IQR squares (%)										
1	20.0	0.11	0.41	1.92	12.51	12.82	13.27	98.0	97.7	97.1
2	40.0	−2.72	−2.56	−0.67	14.86	15.26	14.73	95.2	94.7	94.8
3	80.0	−0.44	−0.35	0.51	5.44	5.72	5.28	93.7	92.2	92.3
Scenario 4-6: Skew-normal distribution with moderate positive skewness (pSN1)										
Overall mean or overall median effect										
4	0.000	0.027	0.014	0.022	0.063	0.063	0.067	93.4	93.3	92.1
5	0.000	0.044	0.021	0.029	0.080	0.074	0.080	90.7	92.1	90.8
6	0.000	0.109	0.029	0.033	0.157	0.121	0.127	86.7	94.0	93.2
I^2 or ratio of IQR squares (%)										
4	20.0	0.34	0.89	2.07	12.76	13.18	13.49	97.6	97.2	96.7
5	40.0	−2.56	−1.84	−0.86	15.08	15.37	14.93	94.0	94.0	94.2
6	80.0	−0.38	−0.25	−0.18	5.79	5.83	5.72	91.1	92.6	92.9
Scenario 7-9: Skew-normal distribution with large positive skewness (pSN2)										
Overall mean or overall median effect										
7	0.000	0.030	0.017	0.025	0.065	0.064	0.069	92.6	92.9	91.2
8	0.000	0.051	0.027	0.034	0.083	0.075	0.081	88.7	91.6	90.1
9	0.000	0.126	0.038	0.041	0.169	0.120	0.124	83.1	93.4	92.8
I^2 or ratio of IQR squares (%)										
7	20.0	0.04	0.54	1.74	12.41	12.81	13.09	98.2	97.7	97.3
8	40.0	−3.71	−2.97	−2.09	15.38	15.56	15.20	94.1	93.8	94.4
9	80.0	−1.12	−1.25	−1.21	6.14	6.23	6.16	91.2	93.4	93.7

RMSE: root mean square error, prob.: probability
NRE: normal random effects model, BC: proposed model using Box-Cox transformation
BC-SI: proposed model using Box-Cox transformation with the sign inversion for negatively skewed data

Table S4 (cont'd). Bias, root mean square error, coverage probability from the two models for scenario of the number of studies $k = 40$.

		Bias			RMSE			Coverage prob. (%)		
Scenario	True	NRE	BC	BC-SI	NRE	BC	BC-SI	NRE	BC	BC-SI
Scenario 10-12: Skew-normal distribution with moderate negative skewness (nSN1)										
Overall mean or overall median effect										
10	0.000	−0.027	−0.032	−0.022	0.062	0.069	0.066	93.6	91.2	92.2
11	0.000	−0.043	−0.039	−0.028	0.078	0.082	0.079	91.3	89.3	91.0
12	0.000	−0.110	−0.061	−0.035	0.157	0.138	0.126	86.8	89.1	93.2
I^2 or ratio of IQR squares (%)										
10	20.0	0.27	0.16	2.01	12.58	12.72	13.29	97.7	97.8	97.0
11	40.0	−2.44	−4.03	−0.73	15.11	15.72	15.05	94.1	94.4	94.1
12	80.0	−0.41	−3.30	−0.20	5.81	7.38	5.74	90.8	91.1	92.8
Scenario 13-15: Skew-normal distribution with large negative skewness (nSN2)										
Overall mean or overall median effect										
13	0.000	−0.031	−0.035	−0.026	0.064	0.071	0.068	92.6	90.0	91.2
14	0.000	−0.051	−0.046	−0.034	0.083	0.086	0.082	89.0	87.5	89.8
15	0.000	−0.126	−0.068	−0.041	0.168	0.139	0.125	83.0	87.5	92.4
I^2 or ratio of IQR squares (%)										
13	20.0	−0.24	−0.38	1.46	12.37	12.53	13.02	98.2	98.0	97.5
14	40.0	−3.63	−5.45	−2.05	15.57	16.24	15.41	93.6	94.0	94.4
15	80.0	−1.34	−5.10	−1.47	6.27	8.65	6.30	90.7	88.4	93.6
Scenario 16-18: Shifted exponential distribution (EXP)										
Overall mean or overall median effect										
16	0.000	0.059	0.039	0.044	0.084	0.074	0.079	85.4	89.3	87.6
17	0.000	0.096	0.053	0.056	0.120	0.089	0.092	76.7	88.6	87.8
18	0.000	0.238	0.082	0.083	0.273	0.145	0.146	61.9	90.4	90.4
I^2 or ratio of IQR squares (%)										
16	20.0	5.69	5.96	6.80	15.83	15.97	16.29	91.9	92.3	91.8
17	40.0	6.47	5.30	5.66	17.55	16.95	16.85	79.9	84.6	84.7
18	80.0	4.22	0.43	0.44	7.63	6.93	6.92	60.3	86.2	86.3
Scenario 19-21: Shifted log-normal distribution (LN)										
Overall mean or overall median effect										
19	0.000	0.012	0.001	0.009	0.058	0.062	0.065	94.8	93.7	93.2
20	0.000	0.034	0.012	0.019	0.075	0.073	0.078	92.4	93.3	92.1
21	0.000	0.208	0.054	0.054	0.250	0.134	0.134	74.4	94.1	93.8
I^2 or ratio of IQR squares (%)										
19	20.0	0.66	1.13	2.44	12.79	13.20	13.58	97.5	97.0	96.6
20	40.0	−0.58	0.03	1.09	14.77	15.14	14.83	93.6	93.1	93.4
21	80.0	5.03	1.86	1.87	8.12	6.47	6.46	55.4	82.8	82.9

RMSE: root mean square error, prob.: probability
NRE: normal random effects model, BC: proposed model using Box-Cox transformation
BC-SI: proposed model using Box-Cox transformation with the sign inversion for negatively skewed data

3 Summary statistics of estimates for the transformation and the shift parameter in the simulation study

Table S5 and Table S6 show summary statistics of estimates for the transformation (λ) and the shift (α) parameter respectively, for each scenario of the number of studies $k = 40$.

Table S5. Summary statistics of estimates for the transformation parameter (λ), for scenario of the number of studies $k = 40$.

Scenario	Random effects distribution	Method	Mean	SD	Min	Q1	Median	Q3	Max
1	N	BC	1.155	0.678	−2.02	0.74	0.86	1.55	4.54
2	N	BC	1.131	0.635	−1.48	0.74	0.86	1.48	4.48
3	N	BC	1.064	0.483	−0.94	0.76	0.86	1.38	4.00
4	pSN1	BC	1.053	0.627	−1.74	0.72	0.82	1.23	4.86
5	pSN1	BC	0.914	0.535	−1.72	0.68	0.78	0.90	4.28
6	pSN1	BC	0.627	0.327	−1.54	0.56	0.66	0.74	2.70
7	pSN2	BC	1.051	0.626	−1.60	0.72	0.82	1.26	4.42
8	pSN2	BC	0.884	0.528	−1.96	0.67	0.78	0.88	4.36
9	pSN2	BC	0.577	0.332	−1.67	0.53	0.62	0.71	2.92
10	nSN1	BC	1.251	0.717	−1.44	0.76	0.88	1.70	4.56
11	nSN1	BC	1.446	0.779	−1.04	0.82	1.19	1.98	5.32
12	nSN1	BC	1.863	0.716	0.36	1.38	1.88	2.36	4.82
13	nSN2	BC	1.262	0.727	−1.30	0.76	0.88	1.72	4.50
14	nSN2	BC	1.485	0.798	−1.48	0.82	1.30	2.02	5.14
15	nSN2	BC	2.029	0.731	0.58	1.58	2.04	2.52	5.00
16	EXP	BC	0.878	0.603	−2.72	0.66	0.78	0.90	4.24
17	EXP	BC	0.586	0.621	−2.57	0.56	0.68	0.80	4.34
18	EXP	BC	0.166	0.608	−2.00	−0.24	0.44	0.56	2.45
19	LN	BC	1.095	0.644	−2.01	0.72	0.84	1.39	4.54
20	LN	BC	0.926	0.549	−2.00	0.68	0.80	0.92	4.60
21	LN	BC	0.186	0.620	−2.08	−0.24	0.47	0.58	2.09

SD: standard deviation, N: normal distribution
pSN1: skew-normal distribution with moderate positive skewness
pSN2: skew-normal distribution with large positive skewness
nSN1: skew-normal distribution with moderate negative skewness
nSN2: skew-normal distribution with large negative skewness
EXP: shifted exponential distribution, LN: shifted log-normal distribution
BC: proposed model using Box-Cox transformation
BC-SI: proposed model using Box-Cox transformation with the sign inversion for negatively skewed data

Table S5 (cont'd). Summary statistics of estimates for the transformation parameter (λ), for scenario of the number of studies $k = 40$.

Scenario	Random effects distribution	Method	Mean	SD	Min	Q1	Median	Q3	Max
1	N	BC-SI	0.726	0.234	−2.02	0.66	0.74	0.82	2.34
2	N	BC-SI	0.736	0.231	−1.87	0.68	0.76	0.82	2.52
3	N	BC-SI	0.753	0.158	−1.28	0.68	0.76	0.82	2.16
4	pSN1	BC-SI	0.727	0.237	−1.74	0.66	0.74	0.82	2.42
5	pSN1	BC-SI	0.713	0.270	−1.72	0.66	0.74	0.82	2.44
6	pSN1	BC-SI	0.607	0.291	−1.54	0.56	0.65	0.74	2.20
7	pSN2	BC-SI	0.722	0.248	−1.60	0.66	0.74	0.82	2.89
8	pSN2	BC-SI	0.702	0.282	−1.96	0.65	0.74	0.82	2.44
9	pSN2	BC-SI	0.566	0.311	−1.67	0.52	0.62	0.70	1.94
10	nSN1	BC-SI	0.727	0.240	−1.66	0.66	0.74	0.82	2.76
11	nSN1	BC-SI	0.711	0.274	−2.04	0.66	0.74	0.82	2.54
12	nSN1	BC-SI	0.610	0.286	−1.68	0.56	0.65	0.74	2.04
13	nSN2	BC-SI	0.728	0.241	−1.65	0.66	0.74	0.82	2.43
14	nSN2	BC-SI	0.702	0.288	−2.54	0.65	0.74	0.82	2.22
15	nSN2	BC-SI	0.562	0.325	−1.88	0.53	0.62	0.70	1.86
16	EXP	BC-SI	0.673	0.356	−2.72	0.64	0.74	0.82	2.32
17	EXP	BC-SI	0.517	0.532	−2.57	0.55	0.68	0.78	2.40
18	EXP	BC-SI	0.165	0.606	−2.00	−0.24	0.44	0.56	1.56
19	LN	BC-SI	0.729	0.237	−2.01	0.67	0.75	0.82	2.34
20	LN	BC-SI	0.711	0.283	−2.24	0.66	0.74	0.82	2.60
21	LN	BC-SI	0.185	0.618	−2.08	−0.24	0.47	0.58	2.30

SD: standard deviation, N: normal distribution
pSN1: skew-normal distribution with moderate positive skewness
pSN2: skew-normal distribution with large positive skewness
nSN1: skew-normal distribution with moderate negative skewness
nSN2: skew-normal distribution with large negative skewness
EXP: shifted exponential distribution, LN: shifted log-normal distribution
BC: proposed model using Box-Cox transformation
BC-SI: proposed model using Box-Cox transformation with the sign inversion for negatively skewed data

Table S6. Summary statistics of estimates for the shift parameter (α), for scenario of the number of studies $k = 40$.

Scenario	Random effects distribution	Method	Mean	SD	Min	Q1	Median	Q3	Max
1	N	BC	1.468	1.011	0.31	0.71	0.87	2.77	3.97
2	N	BC	1.564	1.015	0.36	0.80	0.98	2.87	4.11
3	N	BC	2.175	1.099	0.57	1.34	1.61	3.44	5.41
4	pSN1	BC	1.316	0.956	0.23	0.66	0.81	2.30	3.73
5	pSN1	BC	1.256	0.876	0.31	0.72	0.86	1.16	3.82
6	pSN1	BC	1.350	0.612	0.50	1.04	1.19	1.38	4.23
7	pSN2	BC	1.315	0.956	0.28	0.66	0.81	2.32	3.84
8	pSN2	BC	1.211	0.857	0.24	0.70	0.83	1.09	3.82
9	pSN2	BC	1.261	0.599	0.52	0.97	1.11	1.28	4.03
10	nSN1	BC	1.605	1.043	0.32	0.75	0.94	2.87	4.04
11	nSN1	BC	1.996	1.100	0.40	0.91	1.80	3.09	4.30
12	nSN1	BC	3.506	1.096	0.80	3.37	3.84	4.20	6.34
13	nSN2	BC	1.621	1.048	0.35	0.75	0.94	2.88	4.06
14	nSN2	BC	2.033	1.098	0.46	0.92	2.05	3.10	4.71
15	nSN2	BC	3.629	1.011	0.66	3.49	3.86	4.22	7.24
16	EXP	BC	1.205	0.891	0.24	0.65	0.78	1.16	3.71
17	EXP	BC	1.188	0.846	0.29	0.68	0.82	1.08	3.79
18	EXP	BC	1.479	0.893	0.39	0.89	1.06	1.68	4.26
19	LN	BC	1.374	0.975	0.30	0.69	0.83	2.67	3.87
20	LN	BC	1.295	0.885	0.33	0.74	0.89	1.23	4.19
21	LN	BC	1.609	0.921	0.45	0.98	1.17	2.07	4.05

SD: standard deviation, N: normal distribution
pSN1: skew-normal distribution with moderate positive skewness
pSN2: skew-normal distribution with large positive skewness
nSN1: skew-normal distribution with moderate negative skewness
nSN2: skew-normal distribution with large negative skewness
EXP: shifted exponential distribution, LN: shifted log-normal distribution
BC: proposed model using Box-Cox transformation
BC-SI: proposed model using Box-Cox transformation with the sign inversion for negatively skewed data

Table S6 (cont'd). Summary statistics of estimates for the shift parameter (α), for scenario of the number of studies $k = 40$.

Scenario	Random effects distribution	Method	Mean	SD	Min	Q1	Median	Q3	Max
1	N	BC-SI	0.836	0.450	0.34	0.64	0.74	0.86	3.32
2	N	BC-SI	0.932	0.449	0.36	0.73	0.84	0.97	3.60
3	N	BC-SI	1.479	0.450	0.57	1.23	1.41	1.61	4.70
4	pSN1	BC-SI	0.837	0.459	0.23	0.64	0.74	0.86	3.32
5	pSN1	BC-SI	0.938	0.498	0.31	0.71	0.83	0.97	3.40
6	pSN1	BC-SI	1.322	0.549	0.50	1.04	1.20	1.39	4.69
7	pSN2	BC-SI	0.840	0.467	0.28	0.63	0.74	0.86	3.36
8	pSN2	BC-SI	0.927	0.510	0.24	0.69	0.81	0.95	3.42
9	pSN2	BC-SI	1.246	0.557	0.52	0.97	1.11	1.29	4.02
10	nSN1	BC-SI	0.835	0.454	0.28	0.64	0.74	0.86	3.45
11	nSN1	BC-SI	0.936	0.493	0.29	0.71	0.83	0.96	3.43
12	nSN1	BC-SI	1.314	0.538	0.48	1.03	1.19	1.39	4.67
13	nSN2	BC-SI	0.833	0.456	0.23	0.64	0.74	0.86	3.41
14	nSN2	BC-SI	0.927	0.500	0.28	0.70	0.81	0.95	3.49
15	nSN2	BC-SI	1.250	0.572	0.43	0.96	1.11	1.28	4.26
16	EXP	BC-SI	0.907	0.570	0.24	0.64	0.76	0.90	3.47
17	EXP	BC-SI	1.080	0.734	0.29	0.68	0.82	1.02	3.63
18	EXP	BC-SI	1.478	0.891	0.39	0.89	1.06	1.67	4.53
19	LN	BC-SI	0.837	0.446	0.30	0.65	0.75	0.86	3.53
20	LN	BC-SI	0.955	0.498	0.33	0.72	0.84	0.99	3.56
21	LN	BC-SI	1.609	0.920	0.45	0.98	1.17	2.05	4.13

SD: standard deviation, N: normal distribution
pSN1: skew-normal distribution with moderate positive skewness
pSN2: skew-normal distribution with large positive skewness
nSN1: skew-normal distribution with moderate negative skewness
nSN2: skew-normal distribution with large negative skewness
EXP: shifted exponential distribution, LN: shifted log-normal distribution
BC: proposed model using Box-Cox transformation
BC-SI: proposed model using Box-Cox transformation with the sign inversion for negatively skewed data
